# The FLUKA Code: An Accurate Simulation Tool for Particle Therapy

**DOI:** 10.3389/fonc.2016.00116

**Published:** 2016-05-11

**Authors:** Giuseppe Battistoni, Julia Bauer, Till T. Boehlen, Francesco Cerutti, Mary P. W. Chin, Ricardo Dos Santos Augusto, Alfredo Ferrari, Pablo G. Ortega, Wioletta Kozłowska, Giuseppe Magro, Andrea Mairani, Katia Parodi, Paola R. Sala, Philippe Schoofs, Thomas Tessonnier, Vasilis Vlachoudis

**Affiliations:** ^1^INFN Sezione di Milano, Milan, Italy; ^2^Uniklinikum Heidelberg, Heidelberg, Germany; ^3^EBG MedAustron GmbH, Wiener Neustadt, Austria; ^4^CERN, Geneva, Switzerland; ^5^Ludwig Maximilian University of Munich, Munich, Germany; ^6^Medical University of Vienna, Vienna, Austria; ^7^Centro Nazionale di Adroterapia Oncologica, Pavia, Italy; ^8^Heidelberger Ionenstrahl-Therapiezentrum (HIT), Heidelberg, Germany

**Keywords:** Monte Carlo, simulation, hadrontherapy

## Abstract

Monte Carlo (MC) codes are increasingly spreading in the hadrontherapy community due to their detailed description of radiation transport and interaction with matter. The suitability of a MC code for application to hadrontherapy demands accurate and reliable physical models capable of handling all components of the expected radiation field. This becomes extremely important for correctly performing not only physical but also biologically based dose calculations, especially in cases where ions heavier than protons are involved. In addition, accurate prediction of emerging secondary radiation is of utmost importance in innovative areas of research aiming at *in vivo* treatment verification. This contribution will address the recent developments of the FLUKA MC code and its practical applications in this field. Refinements of the FLUKA nuclear models in the therapeutic energy interval lead to an improved description of the mixed radiation field as shown in the presented benchmarks against experimental data with both ^4^He and ^12^C ion beams. Accurate description of ionization energy losses and of particle scattering and interactions lead to the excellent agreement of calculated depth–dose profiles with those measured at leading European hadron therapy centers, both with proton and ion beams. In order to support the application of FLUKA in hospital-based environments, Flair, the FLUKA graphical interface, has been enhanced with the capability of translating CT DICOM images into voxel-based computational phantoms in a fast and well-structured way. The interface is capable of importing also radiotherapy treatment data described in DICOM RT standard. In addition, the interface is equipped with an intuitive PET scanner geometry generator and automatic recording of coincidence events. Clinically, similar cases will be presented both in terms of absorbed dose and biological dose calculations describing the various available features.

## 1 Introduction

Popularity of Monte Carlo (MC) techniques in the field of medical physics is increasing rapidly in recent years. This is specifically the case for hadron therapy. MC simulations are an essential tool for the design and commissioning of novel clinical facilities, allowing a detailed description of the beam line and the delivery system. They are also widely used for bunker design, shielding, and radiation protection. MC calculations are a valuable tool for the commissioning of Treatment Planning Systems (TPSs). Furthermore, MC codes can represent a unique instrument for validation, and possibly the improvement, of analytical TPS’s. In situations where experimental validation is unavailable and/or analytical methods are inadequate, MC simulation allows patient-specific dose calculation. Aspects where MC techniques can be more effective compared to traditional, analytical methods may be summarized as follows:

- MC methods take into account more realistically the composition of the human body (1–3), with a possible advantage over the water-equivalent approach typically used in analytical TPS’s.
- MC methods naturally include mixed field description and three-dimensional spread of the particle fluence, reliably describing the transport, and the interaction of the primary beam and of the secondaries (4, 5).
- In-beam monitoring of the irradiation through positron emission tomography or the detection of prompt photons from nuclear de-excitation can be performed using MC simulations, taking into full account the complexity of the mixed radiation field and tissue stoichiometry (3, 6–8).

The FLUKA code (8, 9) is a general purpose Monte Carlo code simulating the interaction and transport of hadrons, heavy ions, and electromagnetic particles. It is jointly developed by the European Organization for Nuclear Research (CERN) and the Italian Institute for Nuclear Physics (INFN). It is built and maintained with the best possible physical models in terms of completeness and accuracy. This approach, usually defined as microscopic, allows sound physical bases to be given to each step. Performance is optimized comparing with particle production data at the single interaction level. No tuning whatsoever on integral data, like calorimeter resolutions, thick target yields, etc., is performed. Therefore, final predictions are obtained with a minimal set of free parameters, fixed for all energies and target/projectile combinations. Results in complex cases as well as scaling laws and properties emerge naturally from the underlying physical models and the basic conservation laws are fulfilled *a priori*. Moreover, the microscopic approach preserves correlations within interactions and among the shower components, and it provides predictions where no experimental data are directly available. When needed, powerful biasing techniques are available to reduce computing time. Descriptions of FLUKA models and extensive benchmarking can be found in the literature (a collection of references can be obtained through the website, www.fluka.org).

Physics models of superior quality have extended the use of FLUKA to medical applications. Apart from physics, FLUKA is one of the first general-purpose MC codes, which translates DICOM files into voxel geometry as part of the combinatorial geometry package of FLUKA (10, 11). Recent developments in the user interface [Flair (12, 13)] further expanded the user-base of FLUKA. Features well received by users include the high-level management of the entire simulation process, including geometry generation (supported by interactive editing and versatile display) and material assignment (supported by built-in libraries, which include ICRU and ICRP tissue compositions). Additional functionalities include semi-automated generation of PET scanners and semi-automated recording of coincident events.

In Section 2, we shall review the status of ionization/multiple scattering models in FLUKA, together with the tools for biological dose simulations. The status of proton and ion nuclear interaction models, including fragmentation, will be reviewed, supported by examples of particle production with benchmarks. Section 3 will be dedicated to the application of FLUKA to the techniques for *in vivo* monitoring of hadron therapy. A detailed presentation of the Flair interface in the context of radiation therapy can be found in Section 4. Finally, a review of the current application of FLUKA in two centers for hadrontherapy (CNAO and HIT) will be presented in Section 5.

## 2 Dose and Biological Dose

### 2.1 Charged Particle Interactions in Matter

The most important atomic processes undergone by charged particles when traversing media consist of Coulomb scattering with both atomic electrons and nuclei. The effect of this same basic process is very different for electrons and nuclei because of their difference in mass. Inelastic interactions with atomic electrons are by far the dominant source of charged particle energy losses (also referred to as electronic stopping power), while they give a contribution proportional to the atomic number Z to angular deflections. Elastic collisions with atomic nuclei result in negligible energy losses – usually referred to as nuclear stopping power – but the angular deflection is proportional to Z^2^. As a consequence, angular deflections are associated mostly with scattering on atomic nuclei, but for the lightest elements where the two contributions become comparable.

Energy losses of charged particles are commonly expressed as an average energy loss per unit path length. The slowing down of energetic protons and ions in matter is governed by collisions with the atomic electrons and leads to the characteristic shape of the depth–dose profile of heavy charged particles with a peaking energy deposition, the so-called Bragg peak.

The nuclear stopping power contribution to the total energy loss of protons and ions in the energy range of relevance for therapy is negligible and will not be discussed further.

The implementation of the electromagnetic physics models in FLUKA, which describe continuous energy losses of heavy charged particles, energy loss straggling, delta-ray production, and multiple Coulomb scattering, is briefly described in the following.

### 2.2 Electronic Stopping Power

Electronic stopping powers are computed by FLUKA starting from the Bethe–Bloch (14–16) formalism. Several corrections to the standard formulation have been implemented in FLUKA in the recent years, allowing to obtain the high precision requested for the transport of therapeutic beams. The implementation follows, with modifications, extensions and refinements, the functional forms presented in Ref. (17), complemented by Ziegler (18–20) at the lowest energies.

The formula for the average energy loss of particles much heavier than electrons and with charge *z* can be expressed by:

(1) $$\begin{array}{l} {\left(\frac{\mathrm{dE}}{\mathrm{dx}}\right)}_{0}=\frac{2\pi n_{e}r_{e}^{2}m_{e}c^{2}z_{\text{eff}}^{2}}{\beta^{2}}[\ln\left(\frac{2m_{e}c^{2}\beta^{2}T_{\text{max}}}{I^{2}\left(1-\beta^{2}\right)}\right)-2\beta^{2}+2zL_{1}\left(\beta \right)+2z^{2}L_{2}\left(\beta \right)\\ \;+K_{M}\left(z,\beta \right)-2\frac{C\left(\beta \right)}{Z}-\delta \left(\beta \right)] \end{array}$$

for spin 0 particles and similarly for spin 1/2 particles. *β* is the projectile velocity relative to the speed of light, *n_*e*_* is the target material electron density ($n_{e}=\frac{\rho N_{Av}Z}{A}$ for an element), *I* its mean excitation energy, *M* is the projectile mass, and $\gamma =\frac{1}{\sqrt{1-\beta^{2}}}$ and *T_*max*_* is the maximum energy transfer to a stationary electron, which is dictated by kinematics and given by:

(2) $$T_{\text{max}}=\frac{2m_{e}c^{2}\beta^{2}\gamma^{2}}{1+2\gamma \frac{m_{e}}{M}+{\left(\frac{m_{e}}{M}\right)}^{2}}$$

Contrary to common approximations, all terms in equation (2) are kept in the FLUKA formulation. The “mean exitation energy” *I* is a sort of logarithmic average over all ionization and excitation levels of the target material. FLUKA uses for *I* the values recommended in Ref. (17); however, the user can override them if new experimental data so suggest, as it is the case for water.

The terms *δ*, *C*/*Z*, *L*_1_, *L*_2_, and *K_*M*_* are all corrections to the Bethe–Bloch formalism. *δ* is the so called “density correction,” extensively discussed in the literature, and connected with medium polarization which, in FLUKA, is computed according to (21). *C* is the shell correction, which takes into account the effect of atomic bonds. This correction becomes important at low energies and, in FLUKA, it is extracted from the proton stopping power values reported in Ref. (17, 22) once all other corrections are undone. *z*_eff_ is the projectile “effective charge,” which takes into account the partial neutralization of the projectile charge when its velocity is not much larger than those of the atomic electrons. For sufficiently large velocities, or for very light ions (e.g., protons and alphas) *z_*eff*_* = *z*, otherwise FLUKA makes use of the effective charge parametrizations proposed in Ref. (23); however, with different parameters in order to disentangle the effect of the *L*_1_, *L*_2_, and *K_*M*_* corrections, which were not considered in the original paper.

The code takes into account the *z*^3^, Barkas (24), and *z*^4^, Bloch (25), corrections (indicated by *L*_1_ and *L*_2_) to the first Born approximation according to the formalisms presented in Ref. (17, 26, 27). A further correction *K_*M*_*, which is not commonly included in stopping power calculations but which turns out to be important for medium-heavy projectiles, is associated with the electron-ion Mott cross section (28). The Bethe–Bloch equation is based on the electron-ion scattering cross sections computed in first Born approximation; however, when the *z*α** < < 1 (*α* is the fine structure constant) condition does no longer hold higher order corrections must be applied. The Mott cross section includes those corrections; however, it is mathematically and computationally very complex. In FLUKA, the Mott cross section parameterization proposed in Ref. (29) as further modified in Ref. (30) are used to compute the correction to the average stopping power, as well as the associated corrections to the secondary electron production cross section and to the energy loss fluctuations.

For protons and alphas, the resulting unrestricted electronic stopping power values are fully consistent by construction to those available at Ref. (22, 31) as long as *I* is left unchanged. The FLUKA formalism has been demonstrated to be able to reproduce with high accuracy, and with a unique value of *I* for a given target, experimental data at energies up to several hundreds of MeV/n for ions ranging from protons to uranium, as shown in Section 2.6.

### 2.3 Secondary Electrons and Energy Loss Fluctuations

Fluctuations associated with charged particle energy losses are an important topic since they determine the shape and position of the Bragg peak. Indeed, its location does not correspond to the nominal particle energy but is situated slightly in front. The classical approaches to this problem, the Landau (32) and Vavilov (33) distributions, suffer from several limitations and are of difficult application in Monte Carlo codes (see Ref. (34) for a discussion).

An alternative approach (34) has been devised for FLUKA, which exploits the properties of the cumulants (35) of distributions. The approach can account for an arbitrary threshold for the explicit production of secondary electrons (“*δ*” rays), for arbitrary step-lengths, and for the contribution of distant collisions to energy loss fluctuations, while assuring the exact match of the average restricted stopping power. It also includes the effect of the Mott correction on energy loss fluctuations.

The explicit production and transport of secondary electrons can be described in FLUKA with a user defined threshold as low as 1 keV.

### 2.4 Multiple Coulomb Scattering

An extended model for charged particle transport through the multiple scattering formalism based on the Molière Theory (36–38) has been specially developed for FLUKA (39, 40).

It can be applied from very small to relatively large steps with a remarkable insensitivity of the resulting distributions. It is complemented by the possibility of switching to single Coulomb scatterings, a possibility which was first proposed and implemented in FLUKA.

Examples of the performances of this model when applied to therapy beams and energies can be found in Ref. (41).

### 2.5 Nuclear Interaction Models

As a consequence of nuclear reactions, the intensity of therapeutic hadron beams is attenuated all along the propagation in tissue. It follows that the dose delivered by primary ions is reduced with increasing depth. While nuclear recoils result typically in negligible spatial modifications of the delivered dose, secondary nucleons, particles, and fragments produced in nuclear reactions can considerably affect the spatial pattern of energy deposition and must be carefully taken into account. For proton beams, only target fragmentation is possible. For heavier ions, projectile fragmentation is the most important process leading to the build-up of secondary particles along the penetration depth. Because of the reaction kinematics, projectile fragments travel nearly in forward direction at almost the same velocity as the incident particle. The secondary lower-charge fragments have typically a longer range than the primary beam and give rise to an undesirable dose deposition beyond the Bragg peak. Furthermore, the fragments angular emission can contribute to an additional lateral spread of the beam particularly evident at the distal side of the Bragg peak, where the primary projectiles are stopped and the dose deposition is due to nuclear fragments only. Hence, in the case of heavy ions, nuclear fragmentation reactions are responsible for the deterioration of the physical selectivity in the longitudinal and transversal dimension especially around the Bragg peak region. The amount of fragments produced generally increases with the mass and charge of the primary particle.

The FLUKA nuclear interaction model, called PEANUT (42–45), provides the nuclear environment for hadron, photon, muon, and neutrino interactions from a few MeV up to the energies, for instance, of the CERN Large Hadron Collider. At energies of interest for therapy, PEANUT models the interactions along the steps of a generalized intranuclear cascade (GINC), followed by an exciton based preequilibrium particle emission and by an equilibrium phase. Detailed descriptions of the “fast” reaction stages, as well as comparisons with particle emission data, can be found in the literature (42–46). A combined benchmark on nuclear interactions and electromagnetic interactions is described in Ref. (47). Produced nuclei form a thermally equilibrated system, characterized by its excitation energy. This system can “evaporate” nucleons, or fragments, or *γ* rays, or even fission, to dissipate the residual excitation. Evaporation and fission in FLUKA are based on statistical approaches (42, 48).

For light residual nuclei (A < 16), where the excitation energy may overwhelm the total binding energy, a statistical fragmentation (Fermi Break-up) model is implemented (49–51). The excitation energy still remaining after evaporation is dissipated via emission of *γ*-rays, as will be described in Section 3. Recently, competition of gamma ray emission with particle evaporation has also been implemented. As will be described in the following sections, the low excitation stages of nuclear interactions are presently under strong development.

Reactions initiated by ions are dealt with by different event generators, depending on the projectile energy. The one related to the highest energies (10, 52, 53) is not of interest for therapy applications and will not be described here.

**Figure 1:**
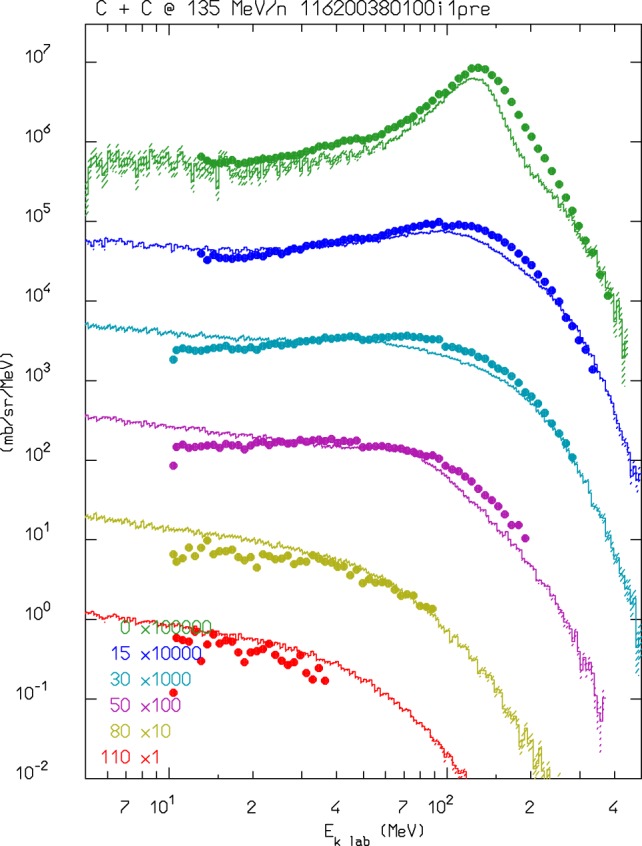
**Double differential neutron production for 135 MeV/n ^12^C interactions in a thin carbon target**. FLUKA–RQMD predictions as a function of neutron energy at several detection angles are shown as histrograms and compared with experimental data (dots) from Ref. (57).

For ions in the few GeV/n energy range and down to ≈0.1 GeV/n, FLUKA uses an interface to a modified version of RQMD-2.4 (54, 55). RQMD is a relativistic quantum molecular dynamics model that can also be run in intranuclear cascade mode. Examples of FLUKA results compared with experimental data when running with the modified RQMD-2.4 model can be found in Ref. (10, 56). Since RQMD provides only the fast stage of the reaction, excited fragments from RQMD are further processed by PEANUT. This allowed also to profit from all the improvements that are ongoing in PEANUT. In Figure 1, the neutrons emitted at en energy close to the projectile one are mainly caused by evaporation. The latest development in the RQMD interface is the inclusion of the preequilibrium stage in the treatment of residual fragments. This stage improves the distribution of high energy ejectiles, in particular for projectile energies in the sub-GeV/n region. An example of the latest performances of FLUKA + RQMD is shown in Figure 1. The agreement is remarkable, especially because RQMD is used at an energy that is at its very limit of application.

**Figure 2:**
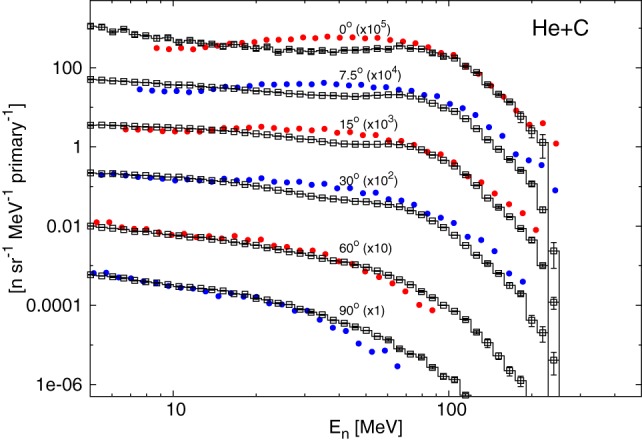
**Double differential spectra of neutrons from 100 MeV/n alphas on a thick carbon target, scaled as indicated at the different angles with respect to the beam direction**. Full circles are experimental data (60), histograms with empty symbols are FLUKA predictions.

The Boltzmann Master Equation [BME (58)] model has been implemented into FLUKA to deal with the lowest energies, below about 150 MeV/n (FLUKA switches gradually between RQMD and BME at threshold). The BME event generator (59) in FLUKA simulates thermalization of a composite nucleus, created in the complete or incomplete fusion of two ions, by sampling from the results of the numerical integration of the BMEs. While complete fusion covers the lowest impact parameter interval, for more peripheral collisions a three body picture of the reaction is implemented. At even higher impact parameters, single nucleon mode break-up/transfer is modeled. Recently, the BME event generator has been interfaced with the PEANUT pre-equilibrium module in order to treat the first de-excitation stage of all nuclei for which BME information is not (yet) available. This development is particularly important, for instance, for reactions induced by alpha particles, as shown in Figure 2, where double differential neutron production by a 100 MeV/n ^4^He beam impinging on a thick carbon target is compared to measurements. As for RQMD, the final de-excitation of the remaining equilibrated nucleus is handled by the FLUKA evaporation/fission/fragmentation module.

### 2.6 Comparisons with Depth–Dose Curves and Lateral-Dose Profiles

FLUKA has been intensively benchmarked against depth–dose data and lateral-dose profiles from various accelerators used for research and clinical ion-beam therapy (IBT), which have been typically acquired with different water columns with parallel-plate ionization chambers [depth–dose (61, 62)] and small volume ionization chambers in water [lateral-dose profiles (63)]. As a consequence of its performances, it is used at IBT centers for independent dose verification in phantom and patient geometries (see Section 5) as well as to generate basic physics input data for clinical treatment planning systems tailored to proton and carbon ion delivery with modern beam scanning (64). These latter TPS basic data include MC-calculated laterally integrated depth–dose distributions, depth–dependent parameters of lateral Gaussian distributions fitted on the MC lateral-dose profiles, and MC-generated carbon ion fragment spectra for biological calculations (41, 61, 62, 65, 66). Recently, FLUKA has also been chosen by a commercial vendor as a validation tool and to provide physics input data for their newly developed carbon ion module (67).

**Figure 3:**
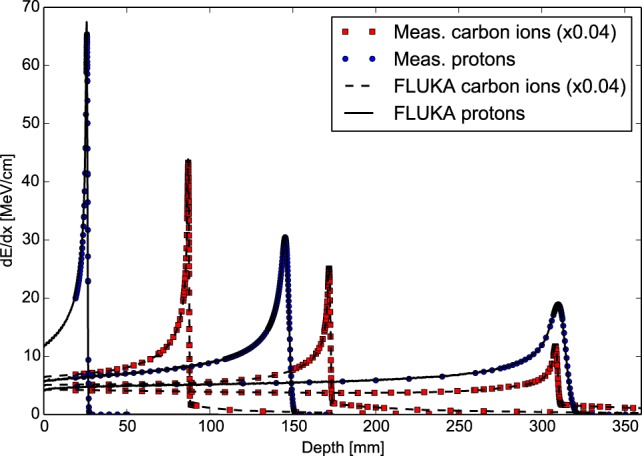
**FLUKA simulations of depth–dose profiles of protons and carbon ions with therapeutic ranges in comparison with measured data at HIT (61)**. The nominal energies before the beamline are 54.19, 142.66, and 221.05 MeV/u forprotons, and 200.28, 299.94, and 430.10 MeV/u for carbon ions.

Figure 3 shows exemplary depth–dose profiles simulated by FLUKA for proton and carbon ions in the therapeutic energy range, compared to measurements taken at the Heidelberg ion therapy center (HIT) with the PeakFinder water column (PTW Freiburg) (61). The nominal energies before the beamline for the presented ions are 54.19, 142.66, and 221.05 MeV/u for protons, and 200.28, 299.94, and 430.10 MeV/u for carbon ions. Since nuclear processes determine notably the shape of the depth–dose profiles, especially for carbon ion and high energy proton beams, these comparisons are not only a sensitive benchmark for the electromagnetic physics models but represent, at the same time, an integral benchmark for the nuclear models in their capabilities of predicting non-elastic nuclear interactions. For different high-accuracy data sets, FLUKA is able to reproduce the position of the Bragg peaks of proton and carbon ion beams with a single ionization potential on average within the experimental uncertainties of about 100 μm. The average dose-weighted dose-difference $\left(\bar{\Delta D/D}\right)$ is below 1% for protons and below 1.5% for carbon ions.

**Figure 4:**
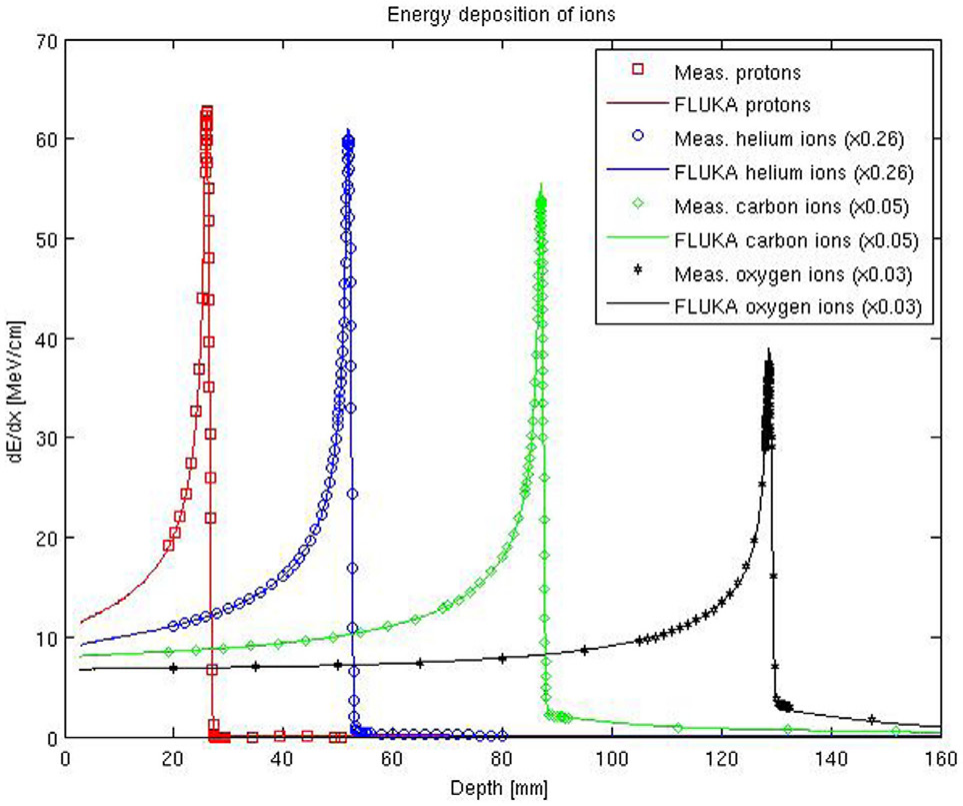
**FLUKA simulations of depth–dose profiles of protons, helium, carbon, and oxygen ions with therapeutic ranges in comparison with measured data at HIT**. The nominal energies before the beamline are 54.19, 79.78, 200.28, and 300.13 MeV/u, for protons, helium, carbon, and oxygen ions, respectively.

An extensive experimental characterization of the other ions available at HIT in comparison to FLUKA simulations is also being performed. A first in-depth characterization of depth–dose profiles of oxygen ion beams has been presented in Ref. (68), and several investigations with helium ion beams are ongoing for both mono-energetic and spread-out Bragg peaks. Figure 4 shows the comparisons between depth–dose profiles acquired with the above mentioned PeakFinder and FLUKA simulations for the different ions available at HIT and different initial beam energies spanning the whole therapeutic range. The nominal energies before the beamline for the displayed ions are 54.19, 79.78, 200.28, and 300.13 MeV/u, for protons, helium, carbon, and oxygen ions, respectively. Quantitative assessment of the level of agreement between measured and simulated depth–dose distributions of these ions has been determined by calculating the weighted chi-square difference for irradiation of an energy yielding the same range (ca. 15 cm in water) without ripple filter, as proposed in Ref. (68). The smaller the weighted chi-square difference is, the higher the similarity is between measurements and simulations. The results indicate for the clinically used protons and carbon ions a level of chi-square agreement of 5.8 × 10^–5^ and 1.1 × 10^–4^, respectively. Compared to this reference level, the helium ions exhibit promising weighted chi-square differences of 2.1 × 10^–4^ and oxygen ions 5.6 × 10^–5^. Additionally, the average dose-weighted dose-difference was evaluated, again for the same energies chosen to provide the same range, and found to be 0.6% for protons, 1.6% for helium ions, 0.8% for carbon ions, and 1.3% for oxygen ions. Again, range agreement within 110 μm could be obtained for both He and O ions over the entire therapeutic energy range with a single ionization potential value in water. Compared to the extensively validated and already clinically used protons and carbon ions, the overall agreement observed for helium and oxygen ions is encouraging, but room for Monte Carlo model improvements is still possible, especially if more experimental data will become available in the therapeutic energy range and for materials of clinical relevance.

**Figure 5:**
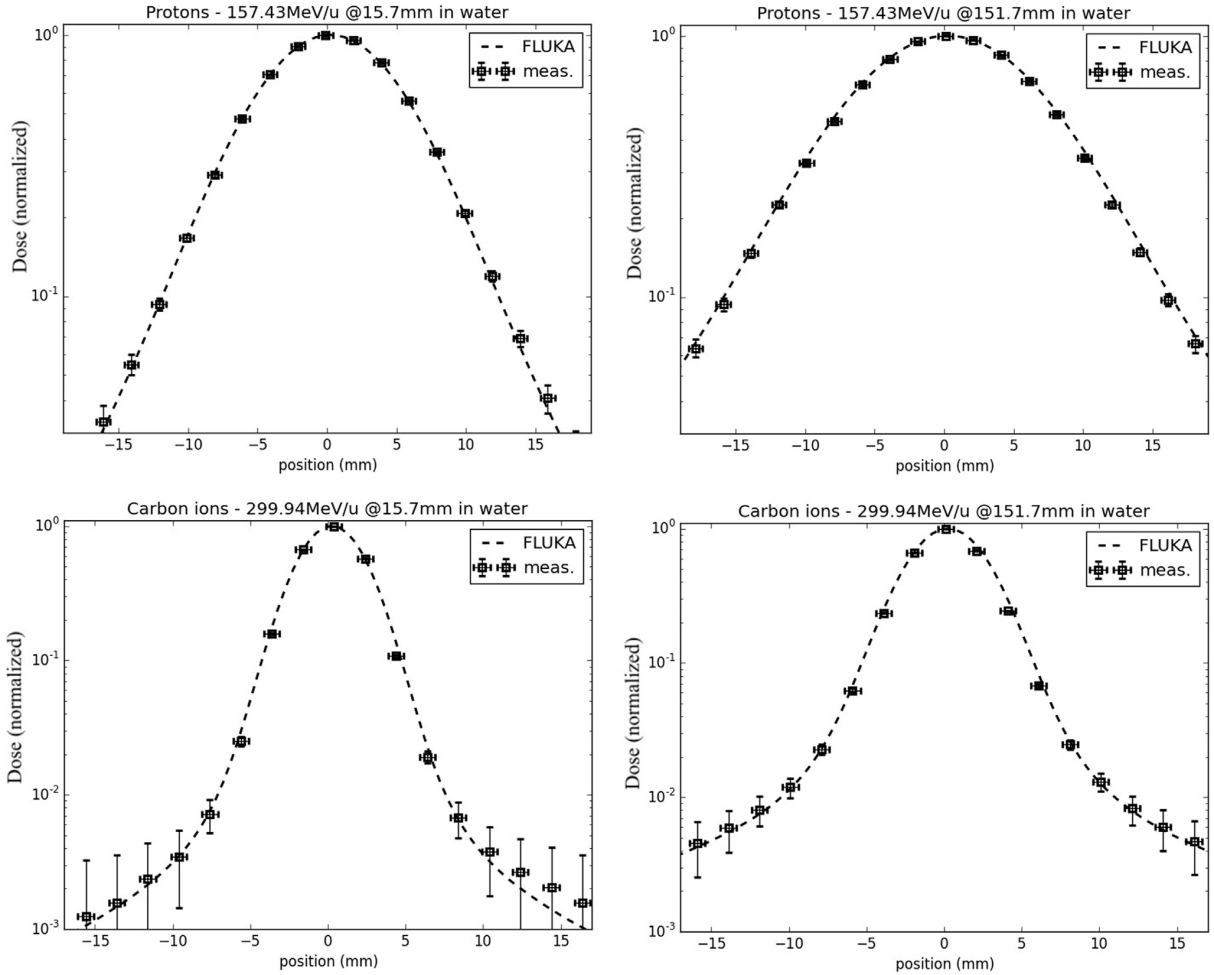
**FLUKA simulations of lateral-dose profiles of protons (top) and carbon ions (bottom) in water, with a nominal initial energy before the beamline of 157.43 and 299.94 MeV/u, respectively, sampled in the entrance region (left) and shortly before the Bragg peak (right), in comparison to experimental measurements taken at HIT**.

In terms of lateral-dose profiles, Figure 5 shows an example of agreement between FLUKA simulations, including the detailed modeling of the HIT beamline according to Ref. (41), and measurements for protons (nominal energy of 157.43 MeV/u before the beamline) and carbon ions (nominal energy of 299.94 MeV/u before the beamline), sampled at two different depths in water in the entrance region, at approximately 16 mm, and shortly before the Bragg peak, at approximately 152 mm. Taking into account unavoidable uncertainties of the measured data in the low-dose region, as well as averaging volume effects of the small cylindrical ionization chambers of 1.5 mm radius in comparison to the dose gradient (63), the agreement is quite satisfactory. A more extensive quantitative comparison of FLUKA simulations and experimental lateral dose-data collected at different energies and depths can be found in Ref. (69).

### 2.7 Biological Calculations

A major rationale for the application of ion beams in tumor therapy is their increased relative biological effectiveness (RBE) in the Bragg peak region, especially for carbon and heavier ions. For dose prescription in carbon ion therapy, the increased effectiveness has to be taken into account in treatment planning while, in proton therapy, a constant RBE of 1.1 is typically applied as recommended by ICRU (70).

**Figure 6:**
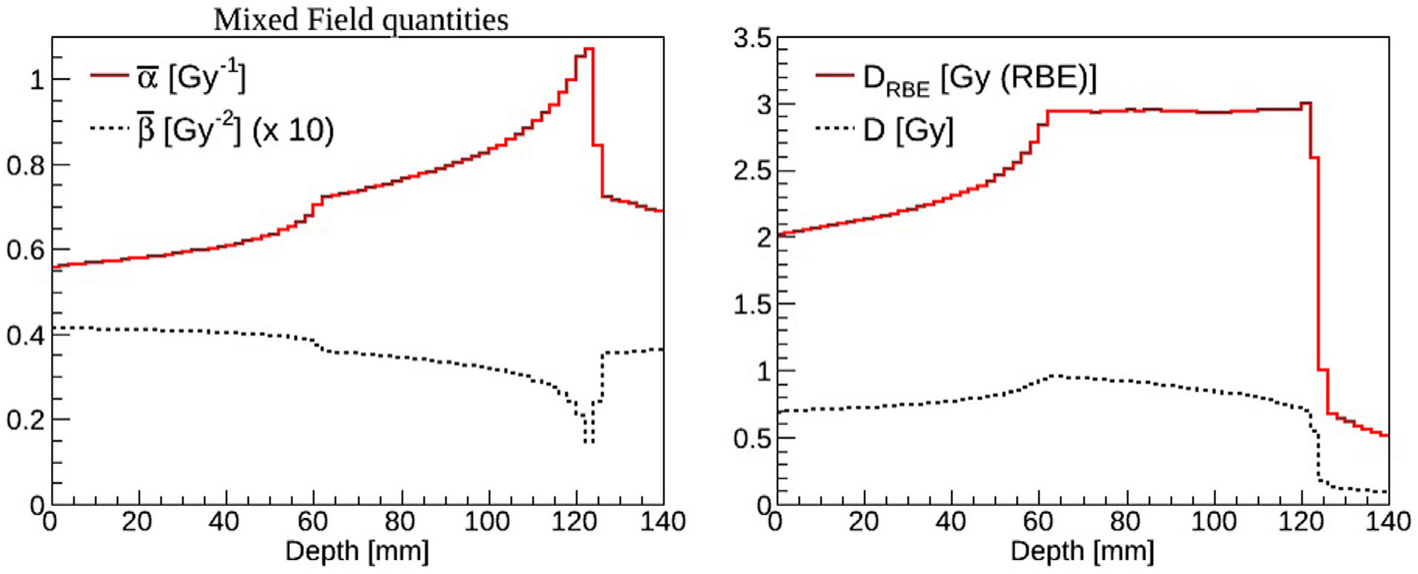
**Left panel: $\bar{\alpha }$ (solid line) and $\bar{\beta }$ (dashed line) mixed radiation field values calculated as a function of depth in water**. The $\bar{\beta }$ results have been rescaled by a factor of ten for display purposes. Right panel: absorbed dose (dashed line) and *D_RBE_* (solid line) values calculated as a function of depth in water.

In order to describe the biological effect with FLUKA, an external radiobiological database has to be integrated. The database can be obtained from experimental data or starting from event-by-event track structure simulations. This approach was adopted in the past to characterize therapeutic proton beams from a physical and biophysical point of view (71, 72). Afterward, the approach has been used in the study of chromosome aberration induction in human cells by neutrons (73). The theory of dual radiation action [TDRA (74)] has been included to describe the non-linear response due to mixed fields, and it has been the basis of more recent calculations interfacing FLUKA with the biophysical model LEM [Local Effect Model (75)], which allows prediction of RBE and RBE-weighted dose (*D_*RBE*_*) distributions in carbon ion beam therapy (4, 66). Starting from these promising works, we decided to develop a general interface within the linear-quadratic formalism (76) available in FLUKA. The users should provide their own biological database in terms of *α* and *β* of different components of the mixed radiation field as a function of energy per nucleon. In order to compute the biological effect, FLUKA applies an approach based on the dose-weighted averages ${\bar{\alpha }}_{j}$ and ${\bar{\beta }}_{j}$:

(3) $${\bar{\alpha }}_{j}=\frac{\sum_{i}\;\Delta d_{i,j}\cdot \alpha_{i,j}}{\sum_{i}\;\Delta d_{i,j}}\text{ and }\sqrt{{\bar{\beta }}_{j}}=\frac{\sum_{i}\;\Delta d_{i,j}\cdot \sqrt{\beta_{i,j}}}{\sum_{i}\;\Delta d_{i,j}}\;,$$

where Δ*d_*i,j*_* is the dose from the *i*-th charged particle (composing the mixed radiation field) with associated *α_i,j_* and *β_i,j_* in voxel *j*, and *i* runs over all particles depositing dose in voxel *j*. RBE and RBE-weighted dose values can be determined for each voxel of the patient knowing the absorbed dose and the dose weighted ave ages ${\bar{\alpha }}_{j}$ and ${\bar{\beta }}_{j}$ [e.g., see Ref. (4)]. As an example in Figure 6, the $\bar{\alpha }$ and $\bar{\beta }$ (left panel) and the absorbed dose and *D_*RBE*_* (right panel) for a carbon ions biologically optimized Spread-Out Bragg peak as available at CNAO are reported. A single-field irradiation plan has been optimized with the CNAO TPS (SIEMENS *syngo*^®^ PT Treatment) to achieve a homogeneous dose distribution of 3 Gy (RBE) in a cubic shaped target (side = 6 cm) centered at 9 cm depth in water. The FLUKA recalculations have been performed for a representative cell line characterized by (*α*/*β*)*_*ph*_* = 2 Gy (*α_ph_* = 0.1 Gy^–1^ and *β_ph_* = 0.05 Gy^–2^) using the same biological database as implemented in the TPS (75). This database is calculated using the radio-biological model LEM I (75), which has been *in vitro* and *in vivo* validated. LEM I is the standard biological model employed at the carbon ion therapy facilities in Europe and has has been developed and benchmarked by the GSI biophysics group.

## 3 *In Vivo* Verification

### 3.1 Introduction

The inverse depth–dose deposition profiles of high energetic proton and ion beams can be used to obtain highly conformal dose distributions for therapeutic purposes. However advantageous, it is then crucial to ensure treatments are delivered with high precision, according to the planner’s prescription. Techniques, which aim to verify the patient geometry as well as the correct treatment delivery before, during, or directly after treatment, have therefore been increasingly investigated in recent years in literature. *In vivo* range monitoring *via *β**^+^-emitter distributions by PET is currently the most advanced monitoring technique routinely used in clinical environments. Simulation studies indicated the feasibility to detect range misses in the order of 6 mm and larger (77), and even better results were reported for favorable anatomical indications in multiple clinical pilot studies with different PET implementations (3, 78–81).

While results achieved with this technique are promising, widespread clinical use of PET monitoring is presently still hampered by several issues. The coincidence measurements of 511 keV annihilation photons allow reconstruction of *β*^+^-emitter maps, which have a complex correlation with the delivered dose. Hence, only a limited correlation between signal on the one hand, and dose and beam range on the other hand, can be achieved. Besides, an extended acquisition time (of the minute-scale) is needed because of the low *β*^+^ activity and relatively small signal-to-noise ratio (SNR) (6). Furthermore, an additional signal attenuation is due to the long delay before starting acquisition after patient irradiation. This delay, of the order of a few minutes, appears in the commonly used in-room or off-line PET monitoring method. Finally, metabolic washout, PET and CT co-registration, and possible patient movements lead to a further decrease of the resolution (79).

The use of prompt-*γ*’s for monitoring was proposed to overcome some of the inherent limitations of the PET technique. Besides a possibly larger signal strength compared to PET resulting in a larger SNR, this method allows in a straightforward way for real-time monitoring and might provide a better correlation of the prompt-*γ* signal with dose and beam particle ranges (82). These potential advantages have yet to be demonstrated in a clinical setting. For such purposes, large efforts have been spent in the design and optimization of detection devices and setups suitable for use in clinics (83–91). Research in this field is still ongoing, as well as feasibility and sensitivity studies aiming at revealing the expected performance and limitations of prompt-*γ* range and dose monitoring (82, 92–95). Monte Carlo (MC) particle transport codes, such as FLUKA (9, 50) are essential tools for such studies.

Using these simulation tools for the above-mentioned applications, implicitly relies on their predictive power, i.e., the accurate description of a range of physics processes relevant to the problem. Dose distribution predictions with MC codes were shown to largely satisfy clinical needs in proton and carbon ion treatment planning a given geometry (61, 96). On the other hand, the development and validation of MC codes for prompt-*γ* production is significantly less advanced. This is partly due to the large complexity of non-elastic nuclear reactions.

Some recent studies have been aimed at elucidating and comparing the predictive capability for prompt-*γ* production of proton and ion beams of some MC and nuclear reaction codes (95, 97–99). Independently from the general agreement about the scarcity of available experimental data, various modeling approaches and measurements have been reported to differ by a factor of 2 to 12 in prompt-*γ* emissions. In particular, differences in the predictions of the distal fall-off positions up to a few millimeters as well as remarkable discrepancies in the relative shapes of the prompt-*γ* profiles are noted. These findings clearly highlight the need for further development and validation of physics models in order to predict prompt-*γ* yields of high energy ion beams, based on a solid measurement database.

This section presents a set of physics models describing prompt-*γ*-emission and *β*-emitter production as a result of non-elastic nuclear collisions of proton and ion beams. Newly developed and refined models are described, which account for discrete and continuous components of *γ* emission spectra including Doppler effect.

The performance of the models for applications to imaging is evaluated using cross section and thick target data.

### 3.2 FLUKA Model Developments for *In Vivo* Verification

The accuracy of physics models included in particle transport codes is of great relevance, especially as their importance in particle therapy has steadily increased over the last years (5). Both *β*^+^ emitters and prompt *γ* production occur in the very last stage of nuclear interactions, therefore they are sensitive to the details of all the reaction “history.”

The relative production probabilities of different residual nuclei are influenced by the exact amount of excitation energy left in the system, by the exact balance of binding energy, but also, and this is more difficult to simulate, by the level structure of the excited and residual nuclei. Not only the level energies but also spin and parity have an influence on isotope production and photon emission. This is particularly true in the Bragg peak region, where the available projectile energy is barely sufficient to initiate the reaction. Low energy nuclear models in FLUKA have undergone a steady development with a particular attention to processes of interest for hadron therapy.

**Figure 7:**
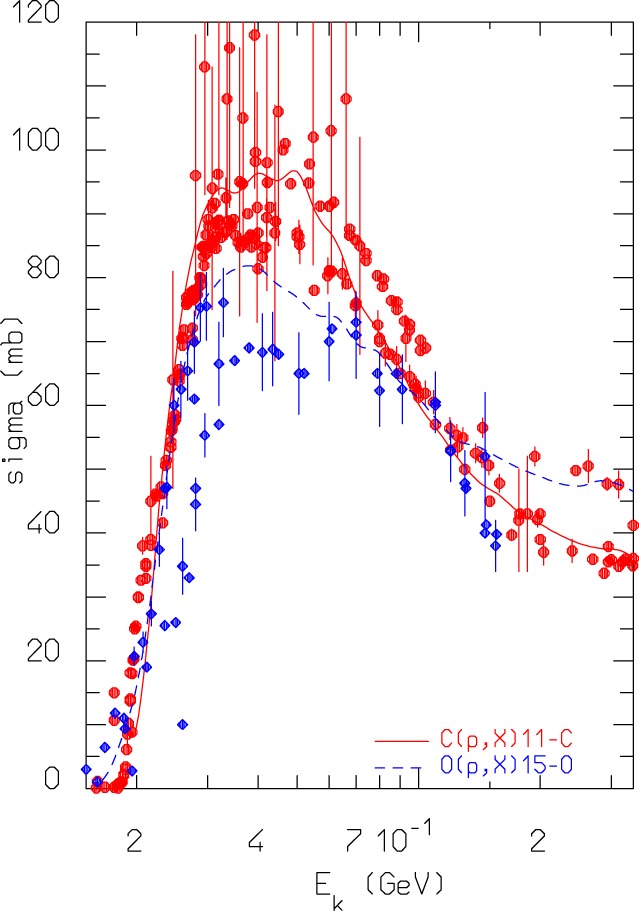
**FLUKA predictions for the reactions *^nat^*^,12^C(*p*,*x*)^11^C and *^nat^*^,16^O(p,x) ^15^O cross sections as a function of projectile energy, compared against data retrieved from the EXFOR library (101)**.

The most important reactions for PET monitoring of proton therapy are ^16^O(p,x)^15^*O* and ^12^C(p,x)^11^C (98). They can proceed through emission of either independent nucleons or deuterons. The emission of composite ejectiles, like d, t, ^3^He, and *α*, is described in FLUKA by the coalescence algorithm in the first stages of the reaction, and by evaporation of fragments in the equilibrium stages. Coalescence is a postemission process, meaning that all combinations of unbound nucleons are checked and the possible formation of light fragments (up to mass 10) is decided based on phase space closeness at the nucleus periphery. This approach works reasonably well at medium/high energies. However, at energies below a few tens of MeV, where binding energies play a crucial role, coalescence is increasingly ineffective in reproducing the data. Recently, a direct deuteron formation mechanism, where the deuteron is formed before being emitted, has been implemented in FLUKA. This mechanism greatly improved the predictive power for reactions, such as (p,d). An example outlining the effectiveness of the new approach and directly relevant for proton therapy monitoring with PET is given in Figure 7. The level of accuracy reached allows to overcome the previously stated need (100) to convolute simulated fluxes with cross section data.

An interesting verification of FLUKA predictions against experimental data taken with a prototype PET system can be found in Ref. (102). For what concerns ion beams, the availability of experimental data on *β*^+^ emitter is scarce, more would be needed to perform a careful evaluation of model predictions. However, the results presented in Table 1 on carbon–carbon interactions at low energy show a reasonable agreement, within 25%, for the production of ^11^C. Indeed, the early work described in Ref. (103) already showed a satisfactory agreement between data and simulations on a full phantom and PET setup.

**Table 1:** **Isotope production cross sections (in millibar) for the fragmentation of 86 MeV/n ^12^C ion projectiles on a carbon target**.

Isotope	Exp.	FLUKA (%)
^11^C	43	54 ± 1
^10^C	4	2 ± 7
^11^B	42	54 ± 1
^10^B	28	25 ± 2
^8^B	2.4	3 ± 6

*Data (104, 105) are compared to FLUKA predictions, integrated over the measured angular range from 2° to 22°. The experimental uncertainty is on the order of 10% (106)*.

Finally, it has to be reminded that electromagnetic models can also play a role in the reproduction of PET reality. A correct reproduction of positron slowing down before annihilation is of course mandatory, but FLUKA goes further in precision and includes an accurate reproduction of the effects of electron binding energy and orbital motion on the emitted photon pair. The resulting acollinearity of the photon pair has been favorably compared with experimental data in Ref. (107).

At the end of the nuclear evaporation stage, the PEANUT model dissipates the residual excitation energy through emission of cascades of *γ* rays. Whenever possible, photon energies and branching ratios are sampled according to a database of known levels and transitions, derived from the most recent release of the RIPL (108) data provided by IAEA. The evaporation stage is also constrained to proceed through known levels when they are available. A first attempt to account for the angular distribution of emitted photons has been implemented, following the formalism in Ref. (109).

Whenever the level compilation is non-existent or incomplete, photon energies are sampled according to a statistical/rotational model that has been validated in the past (110).

**Figure 8:**
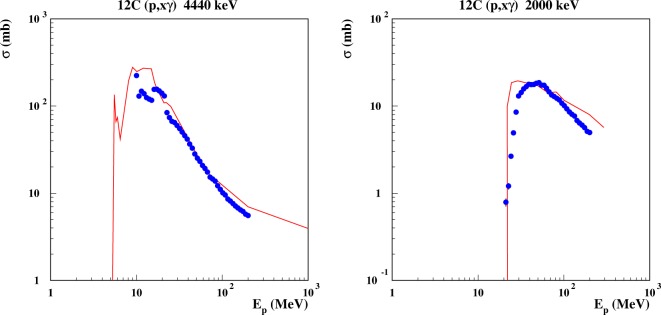
**Excitation function for the emission of discrete *γ* lines from proton-induced reactions on carbon**. Left: the 4.440 MeV line, corresponding to the de-excitation of the 1st excited level in ^12^C, the 2nd excited level in ^11^B, the 2nd excited level in ^11^C. Right: the 2.0 MeV line, from the 1st excited levels of ^11^C and ^11^B. Curves are FLUKA predictions, dots are evaluated data from Ref. (111).

The most stringent requirement for a model of prompt photon production is the capability to reproduce excitation functions of single *γ* lines. Those depend on the capability to reproduce both the branchings in the various reaction channels, and the *γ* de-excitation flow. Examples of such excitation functions for proton-induced reactions in carbon are shown in Figure 8, where FLUKA results compare favorably with experimental data (111).

### 3.3 Model Comparison with Integral Measurements

Complementary to single interaction data, which can give a direct evaluation of the model performances, the study of integrated data for therapeutically relevant scenarios allows an estimation of the model performance for specific applications, such as conceptual and detector design studies.

#### 3.3.1 FLUKA Configuration and Modeling of the Setups

Several prompt-*γ* measurements using ion beams have been conducted in recent years with the purpose to characterize and quantify *γ* production for therapeutic scenarios. Table 2 lists prompt-*γ* measurements selected for comparison. They span differing experimental setups for proton and carbon ion beams at various therapeutic energies and include polymethyl methacrylate (PMMA) and water targets. In all considered experiments, the beam hits a homogeneous tissue-like target with the detectors positioned in a direction perpendicular to the beam axis.

**Table 2:** **Prompt-*γ* experiments selected for comparison with simulations**.

Setup	Beam	Target	Detector	Facility	Exp. reference
S_I_	160 MeV proton	PMMA	NaI	WPE, Essen	(112)
S_II_	95 MeV/u ^12^C	PMMA	BaF_2_	GANIL, Caen	(95)
S_III_	310 MeV/u ^12^C	Water	BaF_2_	GSI, Darmstadt	(95)

**Figure 9:**
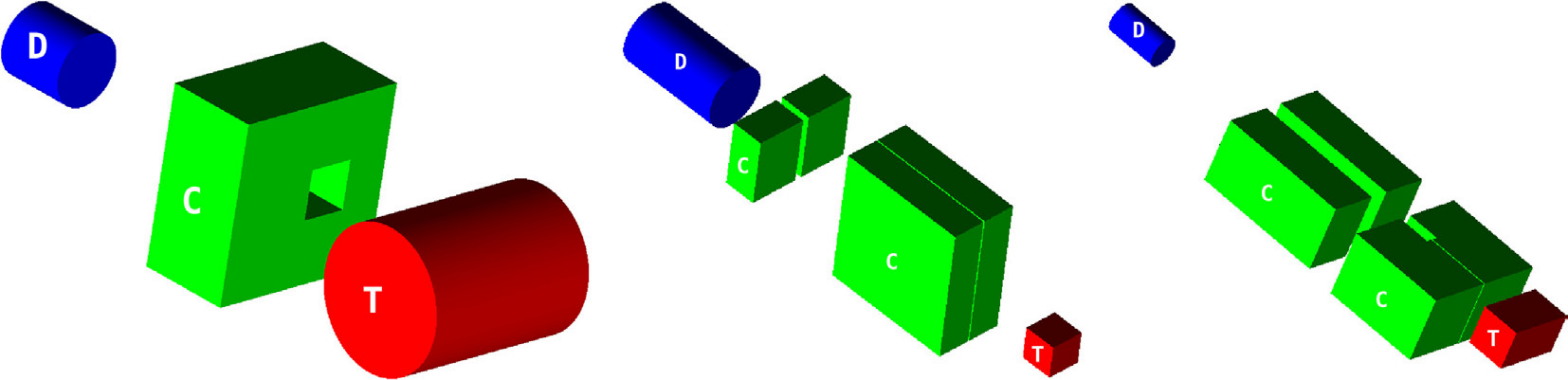
**Simulated configurations of targets (T), collimators (C), and detectors (D) for S_I_ (left), S_II_ (middle), and S_III_ (right), each drawn to scale**. The beam impinges perpendicularly on the center of the target face marked with a ‘T’.

Measured data include *γ* emission profiles with depth as well as photon energy spectra. For each experiment, the relevant measurement configurations and main setup elements were modeled to scale for FLUKA simulations as specified by the experimentalists, including notably: beams, targets, collimators, and detectors (see Figure 9).

Neutrons were the major source of contamination in detectable counts. For noise rejection purposes, S_II_ and S_III_ were both time gated and energy windowed, applying a 2 MeV low-energy threshold. These settings were reproduced for the simulations. Lead collimators were placed between the target and the detector, as shown in Figure 9. The reader is referred to the original references for further experimental details (see Table 2).

The measurement setup S_I_ was repeated in three different configurations: (1) with no collimation device, (2) with a lead block, instead of the collimator, in front of the detector, and (3) with the collimator, as depicted in Figure 9. The dataset is presented as a background-subtracted photon spectrum in two manners. These difference spectra aim to obtain a larger gamma-to-neutron signal ratio in the experimental data. The first one, termed “opening difference” in the following, is the difference of the spectra for the opened (3) and the closed (2) collimator configuration. The second one, termed “wall difference,” is the difference of the spectra acquired for the no-collimator (1) and the closed-collimator (2) configuration. The geometry shown in Figure 9 (left) is for a target-to-detector distance of 50 cm. A separate dataset for target-to-detector distance 100 cm was also acquired (112). The detector, a NaI crystal, was determined to have a resolution of 7% full width at half maximum (FWHM) at 662 keV (112). The simulated energy spectra were therefore convoluted with the Gaussian distribution reflecting the measured detector resolution.

Certain experimental details, which are not accounted for by the simulations, may have an effect on the experimental data, causing artifacts. These potentially include ghosting from preceding beam pulses as well as geometry, setup, and reconstruction details, which are not reproduced by the simulations.

#### 3.3.2 Prompt-Gamma Energy Spectra

**Figure 10:**
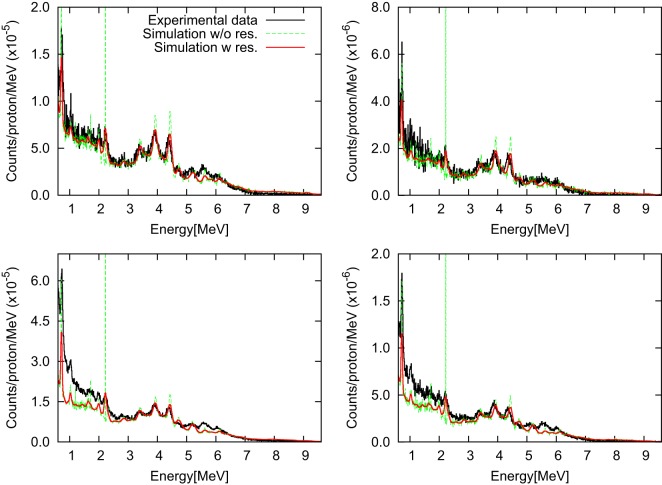
**Background-subtracted photon energy spectra for setup S_I_ with target-detector distances of 50 cm (left) and 100 cm (right) are shown for the “opening difference” (top) and the “wall difference” (bottom)**. Simulated spectra with and without intrinsic detector resolution are presented in comparison with measured data (112).

Simulations of photon spectra resulting from proton beams are presented in Figure 10 in comparison with measurements for setup S_I_ with and without accounting for the intrinsic detector resolution. Data are presented as background-subtracted photon spectra for the configurations “opening difference” and “wall difference.” Measured and simulated spectra are normalized to the number of primary protons and the energy bin, originally 9.83 keV.

Overall, the agreement between simulated spectra and experimental data is excellent. In particular, an agreement within about 10% is found for the “opening difference”-spectra. For the “wall difference”-spectra (difference between closed-collimator and no-collimator configurations), the accuracy of the simulation is also favorable, achieving an agreement within 10% for energies beyond 2 MeV. The result is remarkable, considering that the “wall difference” configuration is more sensitive to measurement artifacts not accounted for by the simulation, such as activation produced by previous beam pulses, pile-up of low energy particles, and scatter-radiation from the nozzle, which is partly screened by the collimator.

#### 3.3.3 Integral Prompt-Gamma Yields as a Function of Depth

**Figure 11:**
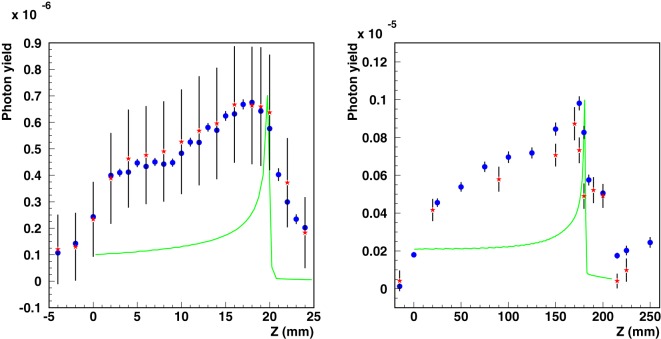
**Integral photon yield as a function of depth**. Simulated (blue circles) and measured [red asterisks (95, 114)] data are shown for carbon ion beams for setup S_II_ (left, 95 MeV/n on PMMA, the original experimental data have been re-evaluated in 2012) and S_III_ (right, 310 MeV/n on water). Simulated depth–dose distributions are also shown as green lines with arbitrary normalization.

The validation of the code in depth profile experiments is essential for prompt-*γ* studies. Figure 11 shows simulations and measurements of photon-depth profiles resulting from carbon beams for setup S_II_ and S_III_. Note that the measured data, previously reported in Ref. (95), have recently been revised by the authors, providing a new absolute normalization (113, 114) and revision of the systematic uncertainties. Hence, updated experimental data are presented in Figure 11. The initial experimental data points, measured at −2 and −4 cm for setup S_II_ and −1.5 cm for setup S_III_, are taken at depths before the start of the phantoms. Only a very small photon yield in air is expected for these positions. The measured signals for these depths can therefore be assumed to represent mostly measurement background. Hence, a background subtraction using the revised data of 6.3 × 10^–7^ (S_II_) and 4.0 × 10^–7^ (S_III_) counts/primary ion has been additionally performed, in order to obtain measured photon yields close to zero for measured data points before the start of the phantoms. A smearing due to detector resolution has been applied to simulated data. For the measured data points, vertical bars indicate the systematic uncertainties as reported in the original papers. For the simulated data points, vertical bars indicate the statistical uncertainty.

By introducing the corrections discussed above, the comparisons show not only a satisfactory agreement in the relative shapes of the profiles but also a good absolute agreement for setups. These findings are consistent with the expected agreement from the comparisons of the prompt-*γ* energy spectra in the previous section.

## 4 Flair and Its Applications to Radiation Therapy

### 4.1 Introduction

**Figure 12:**
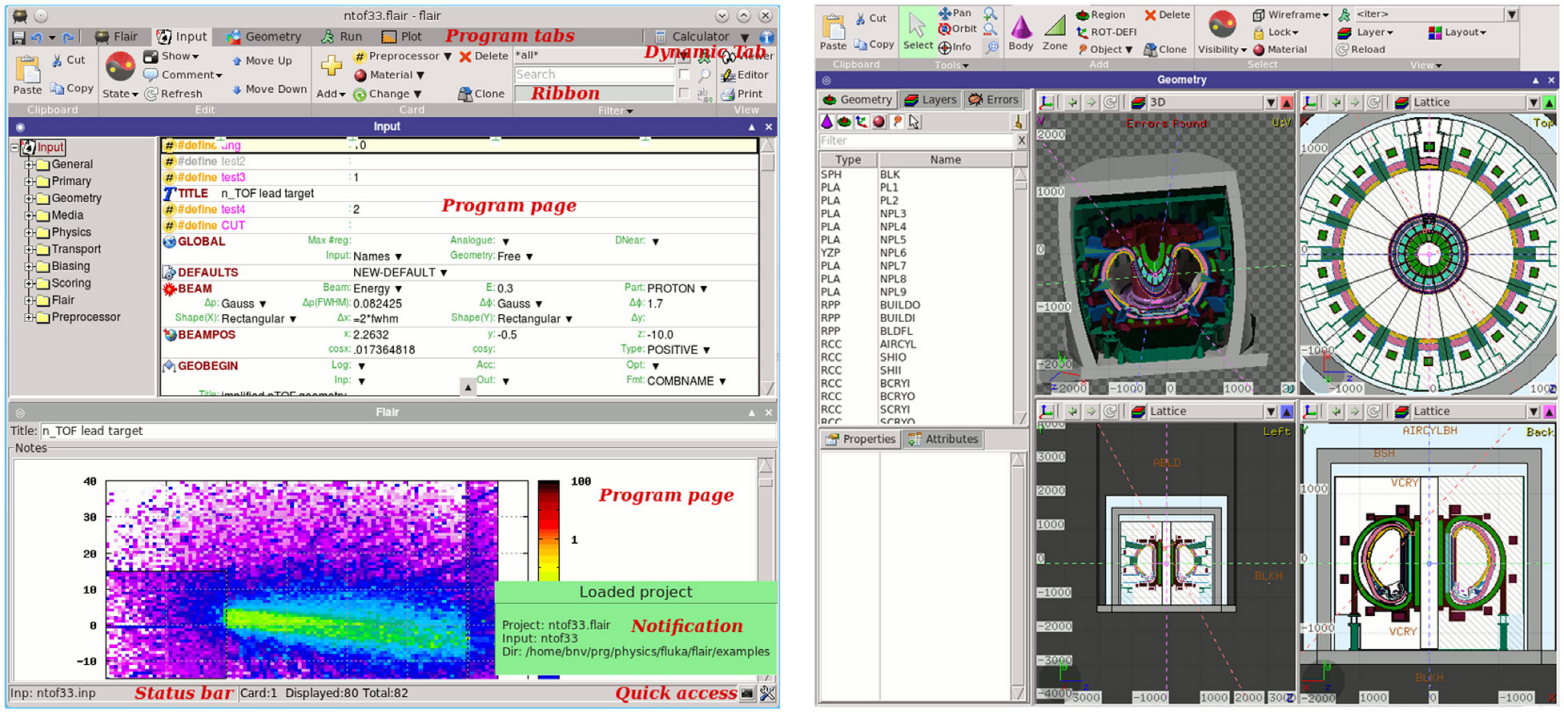
**Flair graphical interface (left), graphical geometry editor (right)**.

Flair (12, 13) (Figure 12) is a user-friendly graphical interface for the FLUKA (8, 9, 50) Monte Carlo transport code. It provides an Integrated Development Environment (IDE) for all stages of FLUKA simulations, from building an error free input file, to debugging, creation of user written routines, execution, status monitoring, data processing, and plot generation. The program employs a custom 2D/3D fully functional graphical editor (13) and debugger for building geometries.

The graphics editor provides very fast graphics with real-time 3D ray-traced rendering of complex geometries as well a dynamic layer mechanism allowing the user to fully customize and create sophisticated views overlaid on the geometry. The use of the program greatly enhances the productivity of the users and provides much steeper learning curve for the beginners. Thanks to the modular design of Flair, recently it was enhanced with the possibility to import, display, process, and convert DICOM files to FLUKA compatible input, as well as with an automatic PET geometry generator. The geometry generator eases the construction of a PET detector with general parameters. The user can also benefit from multiple templates of commercial PET scanners provided within the interface. This section describes the current state of implementation of the medical tools already functional inside Flair as well the future plans. The program and the source code can be freely downloaded from Ref. (115).

#### 4.1.1 DICOM Description

Digital Imaging and Communications in Medicine (DICOM) (116) is a standard for handling, storing, printing, and transmitting information in medical imaging. DICOM supports a wide range of medical images across the fields of radiology, cardiology, pathology, and dentistry. The format is quite versatile and can host practically any kind of information.

Depending on the modality type of each file, a different class has been implemented in Flair to cope with it. Presently, Flair is able to handle the following modalities:

- *CT* – Computed tomography, represented with multiple files each containing a 2D sliced Z image.
- *MR* – Magnetic resonance imaging, with all the necessary information in a single (or multiple files) containing a 3D representation.
- *RTDOSE* – Radiotherapy dose distribution.
- *RTPLAN* – Radiotherapy treatment plan.
- *RTSTRUCT* – Radiotherapy structure set, describing structures/objects.

### 4.2 DICOM *Processing* in Flair

Flair is using pydicom (117), an open source package for reading and writing DICOM files using the python programing language, and the numerical python – NumPy (118) libraries for processing the DICOM files. Pydicom can read and write all standard DICOM files, including nested sequences such as found in DICOM RT files or in structured reports.

**Figure 13:**
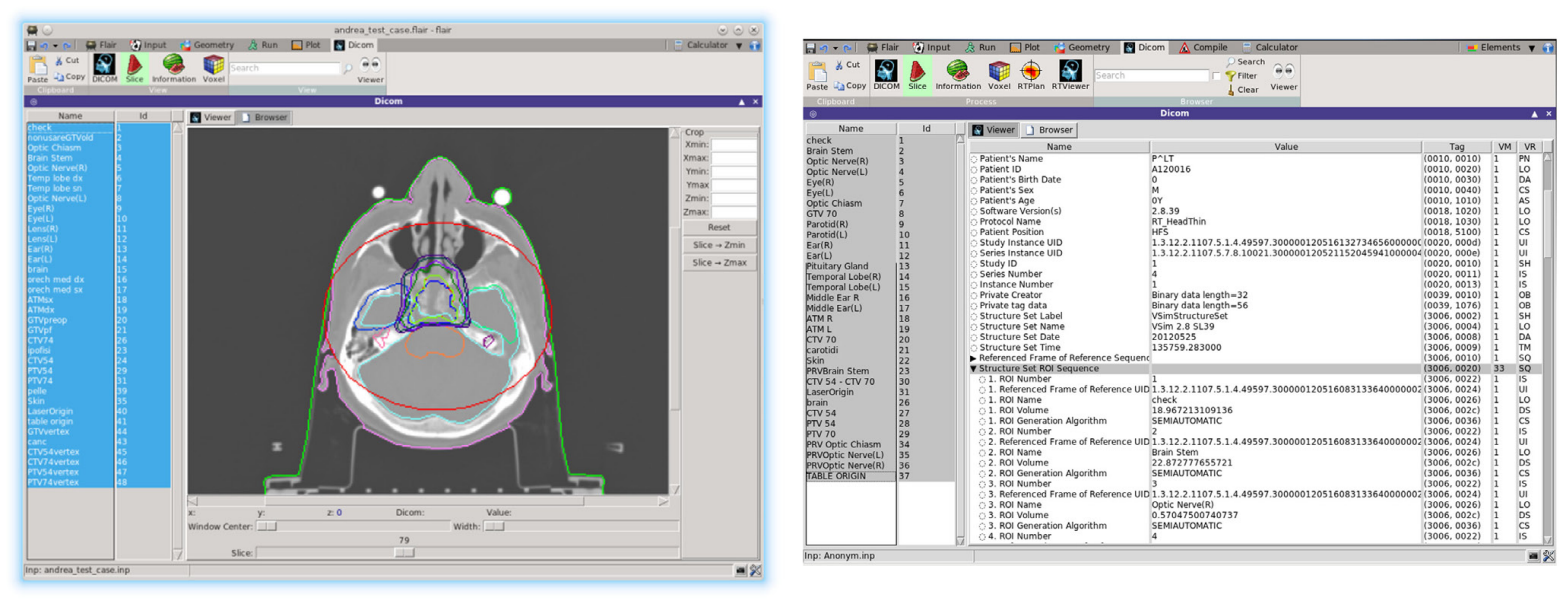
**Flair dicom viewer, with RTSTRUCT superimposed (left); DICOM tree browser (right)**.

The user is able to inspect selected DICOM files either graphically from the Flair DICOM slice viewer or from the enhanced tree structure text browser (Figure 13). The slice viewer is capable of displaying the 2D slices from the DICOM for the CT, MR, RTDOSE, and RTSTRUCT modalities and allows the user to perform simple operations on the slices (like cropping, rescaling, etc.). For RTSTRUCT files, the structures are overlaid on the corresponding display of the CT/MRI slices.

**Figure 14:**
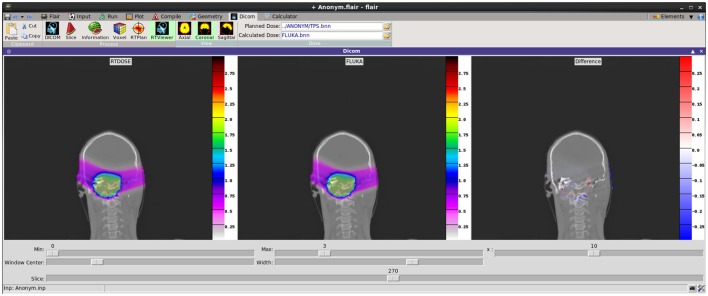
**New DICOM Viewer interface**. Coronal CT plane with mapped physical dose [Gy] from RTDOSE and FLUKA calculation. On the right differences between obtained values [Gy].

Recent development effort was put on providing a better visualization and comparison between TPS and FLUKA re-calculated dose values. The new RTViewer (Figure 14) is able to present 2D cross-sectional CT images for axial, coronal, and sagittal planes combined with the RTDOSE and FLUKA calculations results. It also supports the user with visualization of the differences between MC calculated dose values and TPS prescription. Current development is focused on displaying MR scans, merging three planes with the overlaying ROIs defined in RTSTRUCT, and providing the user with referenced DVH plots.

#### 4.2.1 DICOM to Voxel Conversion

The CT scans contain integer values (so-called *Hounsfield Units*) reflecting the X-ray attenuation coefficient *μ_x_* as a linear transformation of the original attenuation coefficient relative to the one from distilled water at standard temperature and pressure (STP) conditions:

(4) $${\mathrm{HU}}_{x}=1000(\mu_{x}-\mu_{H20})/(\mu_{H20}-\mu_{\mathrm{air}})$$

typically in the range of −1000 ≤ HU ≤ 3500.

Air has typically a HU of −1000, which is the lowest HU value in the file. In FLUKA, we loosely use the word *organ* to indicate a group of voxels (or even more than one group) made of the same *tissue* material (same HU value or in a given HU interval). Internally, FLUKA will handle each organ as a constructive solid geometry region, possibly in addition to other conventional *non-voxel* regions defined by the user.

Assigning a separate material to each of the ~3000–5000 HU values, typically present in a CT, is neither memory- nor CPU-efficient for simulations. Therefore, ranges of HU are grouped into organs while providing a mechanism to allow a continuous HU-dependent scaling of interaction properties of the materials. Flair includes the Schneider (1) parametrization, which segments the CT into 24 materials of defined elemental composition based on the analysis of 71 human CT scans, and assigns to each material a *nominal mean density*, e.g., using the density at the center of each HU interval (1, 119, 120).

*Real density* (and related physical quantities) varies continuously with HU value, therefore in FLUKA, we split the 24 material description in smaller intervals (41 intervals in total), and we apply a scaling correction. Specific ranges of HU values share the same material and during transport an additional scaling factor is applied on the density for the nuclear and for the electronic processes, based on the real HU value. To accommodate for this change, the FLUKA voxel format was enhanced to include the possibility to embed FLUKA input cards that contain all the information on the materials, assignments, and correction factors.

#### 4.2.2 Radiotherapy Treatment Information

As already mentioned, Flair is now capable of importing also the radiotherapy treatment data described in the dedicated DICOM RT (RTSTRUCT, RTDOSE, RTPLAN) standard.

##### 4.2.2.1 RTSTRUCT

The radiotherapy structure set object of the DICOM standard is used for the transfer of patient structures and related data, between the devices found within and outside the radiotherapy department. It contains mainly the information for regions of interest (ROIs) and points of interest (e.g., dose reference points). The ROIs can be used during the simulation for calculating Dose Volume Histograms (DVH) or perform special scoring on an organ basis. The ROIs are represented as the points belonging to a closed polygon using 2D coordinates (not rounded to the pixel size of the corresponding CT image).

When selecting an RTSTRUCT file to be embedded into the VOXEL file, Flair will identify for each voxel to which ROIs it belongs. The case of voxels belonging to more than one ROI is also taken into account. A matrix containing the voxel to ROI correspondence is included in the VOXEL file read by FLUKA. This additional matrix is used by Flair for plotting purposes and/or by FLUKA for scoring and DVH calculations (Figure 13).

Flair provides some checks on the structures like calculating volumes using the true polygonal information or the discretization to voxels. Typical differences up to a few percent can be noticed induced by the quantization process.

##### 4.2.2.2 RTDOSE

**Figure 15:**
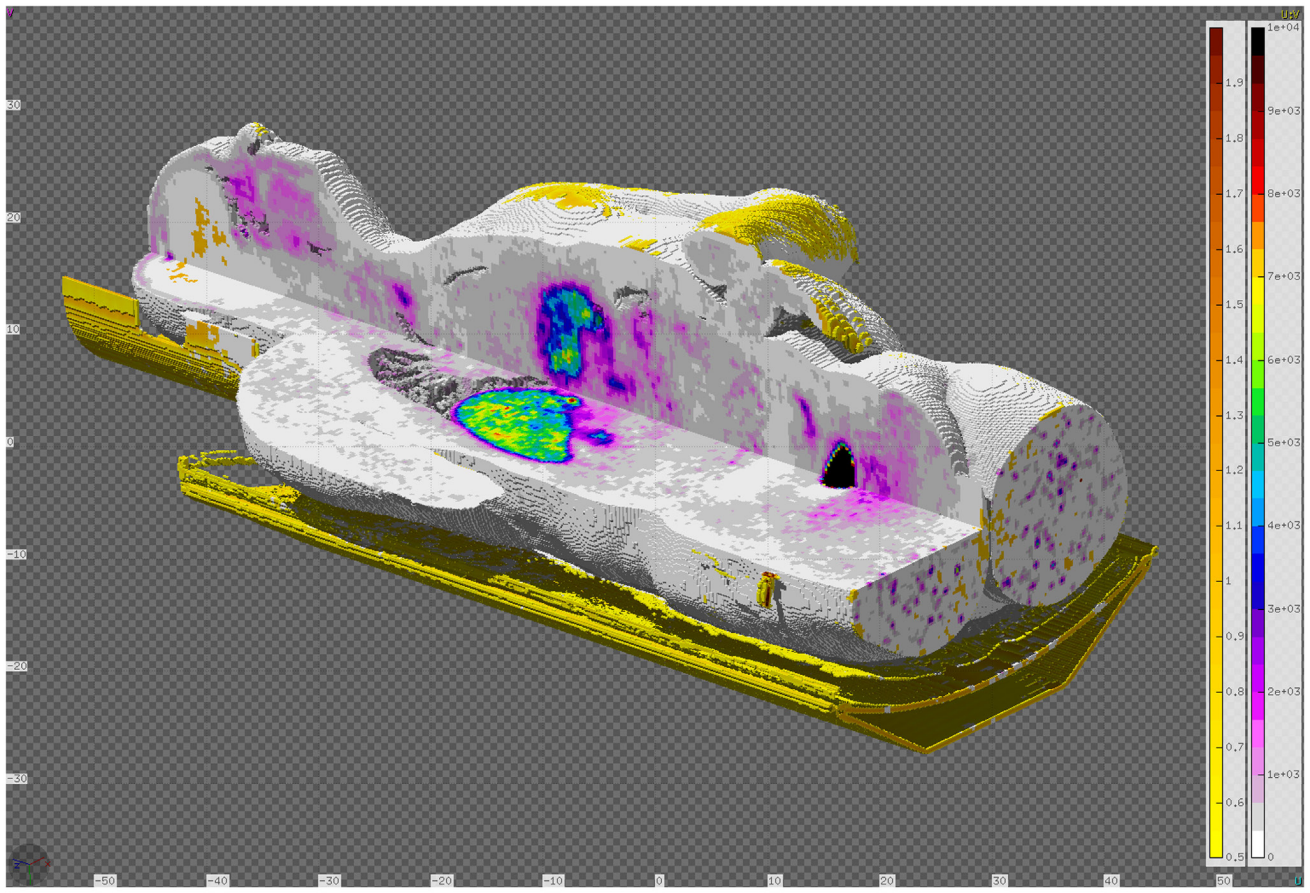
**DICOM to VOXEL import of CT data together with the RTDOSE superimposed**. Displayed using the Flair geometry editor.

The RTDOSE can be converted to a FLUKA USRBIN, a 3D mesh tally. This is possible for all modalities having a PixelData tag like CT, MR, and RTDOSE. Once converted, it can be further used for plotting and comparing the results, e.g., from the output of a treatment planning system with a FLUKA simulation. In the RTViewer (Figure 14), the user can import the chosen sequence dose data or compare the entire treatment fraction and visualize it mapped on the CT scans. USRBIN can be also used as a primary source particles generator, e.g., the PET/CT dose description followed after an FDG (Figure 15).

##### 4.2.2.3 RTPLAN

The RTPLAN contains the information on the treatment plans generated usually by a Treatment Planning System (TPS). Typically such plans provide the information about the treatment fractions, describing the external beams of hadron therapy application. Parameters defined for every single beam spot in the plan are grouped into the beam sequence, where they are enumerated using the control points. Control points checklist includes several information, i.e., particles energy, scan spot position, and number of monitor units. In addition, for each beam sequence, the information for particle type, position of the isocenter according to the DICOM file and gantry, patient and table angles are defined. Flair is currently able to export the most frequently used beam sequence parameters into an external file, from which the special RTPLAN source routine reads and determines the entry source for the FLUKA simulations. While exporting data, Flair performs validation checks on the DICOM file using available control variables.

When defining the beam spot position, RTPLAN refers to its own coordinate system – *Gantry Coordinate* system and *Isocenter Position*. Flair is able to apply correct rotations and translations to the VOXEL structure, and as a result updates the FLUKA input file in order to prepare the fully functional ready-to-run simulation for each single beam field. Further, postprocessing enables to combine simulation beam sequences outputs to one fractional dose file and to visualize it in RTViewer. Current work is focusing on importing less frequently used RTPLAN parameters and simplifying the entire process of treatment plan re-simulation.

### 4.3 PET Scanner Simulation Tools for FLUKA

PET is a commonly used imaging technique, based on detecting in coincidence the pair of annihilation photons created from the decay of a *β*^+^ emitter. Such positron-emitter nuclei are traditionally inoculated to the patient by means of a radio-pharmaceutical drug, in order to analyze the metabolic activity of the body tissue and in the hope of detecting hints of unusual behavior from tumor cells. As an example, Fludeoxyglucose or 18F-FDG is a glucose-analog radio-pharmaceutical, where a normal hydroxyl group is substituted by the ^18^*F* radioactive isotope. This substance is used to study the glucose consumption of the cells.

Apart from its traditional use in nuclear medicine, PET is nowadays the only clinically available method for a non-invasive monitoring of the dose delivery for hadron therapy. However, as mentioned in Section 3.1, there are still important concerns when using commercially available PET scanners for proton or ion beam treatment monitoring, usually compelling to drastically redesign them. Monte Carlo codes are thus crucial to evaluate the performance of new PET prototypes and are an essential tool to infer the dose map from the positron-emitter distribution. To ease the simulation of full PET scanner simulations with FLUKA, taking advantage of the latest developments on the models for beta-emitter production presented in Section 3, Flair incorporates a dedicated PET scanner tool, which covers all the steps from the creation of the geometry of the PET ring to the reconstruction of the image from the coincidence events.

#### 4.3.1 Building the PET Geometry

With the aim of covering most of the collinear pairs of annihilation photons, the geometry of PET scanners is generally composed of an array of detector scintillators, describing a complete or partially opened cylindrical structure.

**Figure 16:**
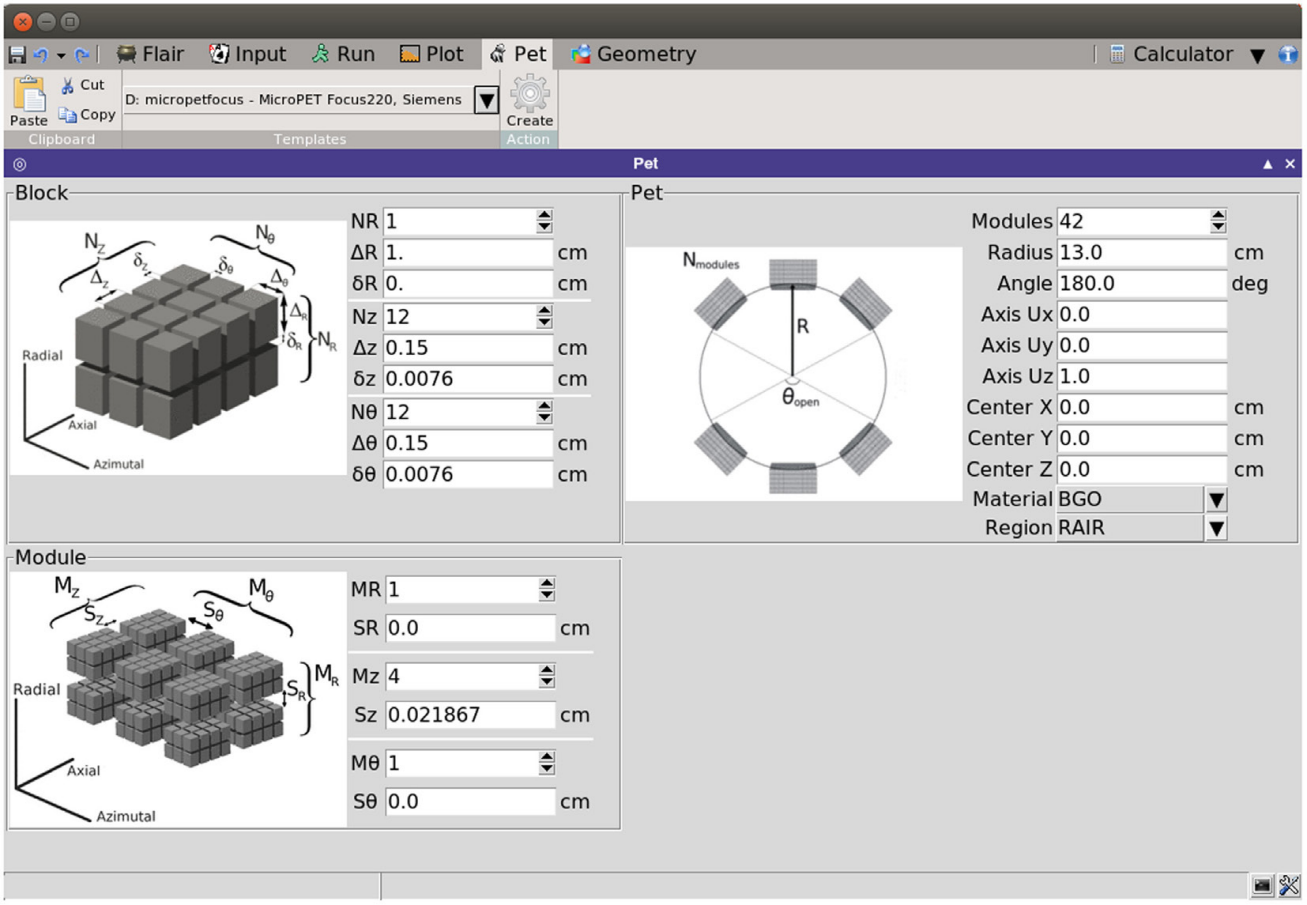
**Flair interface of the PET geometry tool**.

Consequently, PET detectors can be built by replication of simple rectangular parallelepiped sub-units. The PET geometry tool exploits the replication capabilities of the FLUKA code (through its LATTICE cards) to generate a PET detector based on few simple geometrical parameters. The interface of the tool is intuitive, with illustrations that give a visual explanation of the meaning of each parameter (see Figure 16).

The natural cylindrical coordinate system of PET scanners is exploited by associating the (*R*, *θ*, *Z*) coordinates with the *(radial/depth, azimuthal, and axial)* coordinates. The interface divides the required parameters in three levels:

- *Block level*, defined as the array of basic scintillators. The parameters that define the block are the number of scintillators in each direction, the elemental detector dimensions, and the separation among contiguous scintillators.
- *Module level*, taken as the array of blocks. The parameters for the module are the number of blocks in each direction coordinate and the separation among neighboring blocks.
- *Ring level*, which defines the global properties of the PET. The parameters are, then, the number of modules along the ring, the radius of the ring, the vector defining the axis of the scanner, the center coordinates of the scanner, the material of the scintillator, the surrounding region where the scanner will be located and the opening angle, in case a partial ring is required.

The replication of the modules along the ring can be structured in partial or full rings, controlled by the opening angle *θ_open_*, which ranges from 0° to 180° (complete ring). The incorporation of partial rings is interesting for “in-beam” PET, where the scanner has to be integrated with other elements at the irradiation room (121). Based on the azimuthal dimensions of the module and *θ_open_*, the interface is capable of estimating the maximum number of modules that could fit in the available space.

**Figure 17:**
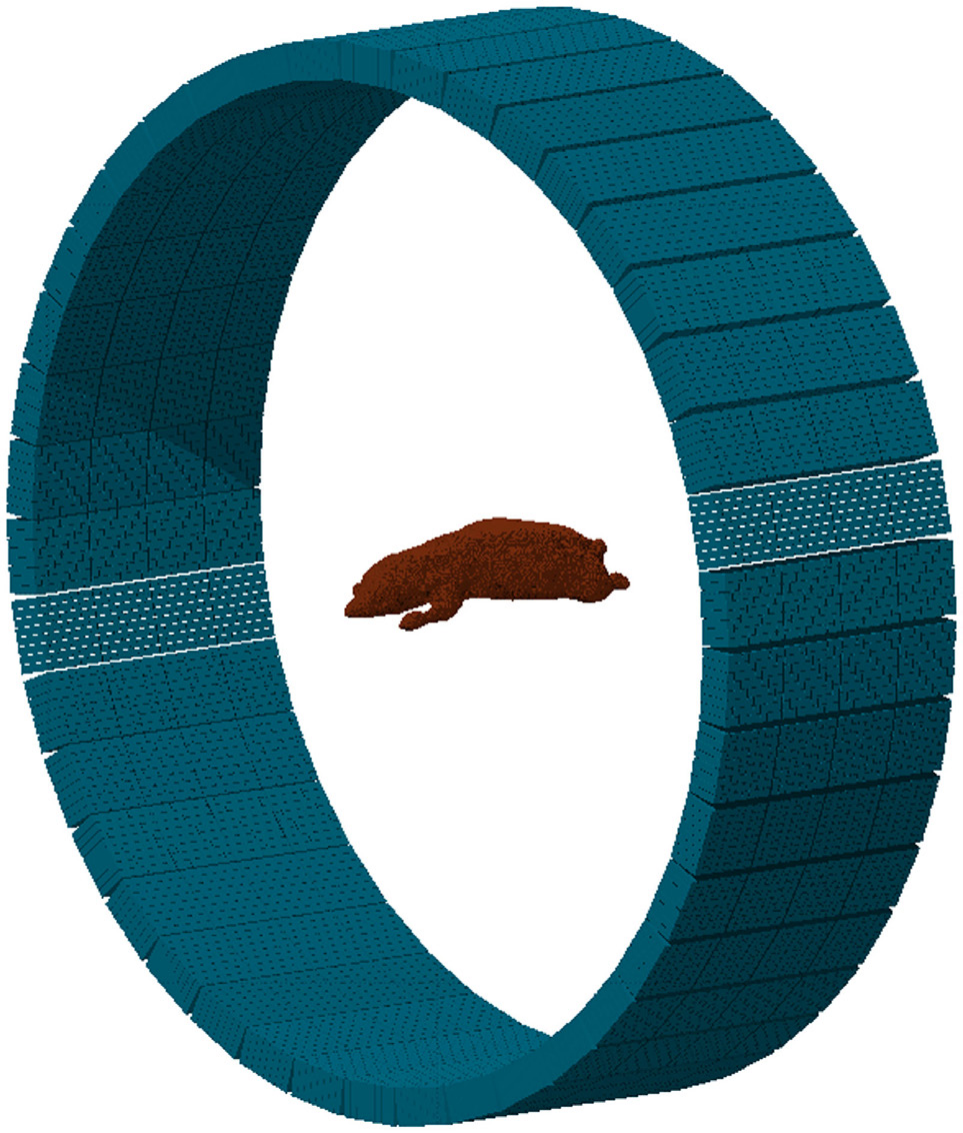
**Example of a MicroPET P4 scanner built with the PET geometry tool**. The phantom is a high-quality, segmented mouse phantom (122, 123), imported to FLUKA voxels with the DICOM tool.

From the previous parameters, the tool generates the input and geometry files (see Figure 17) providing the basic cards for a FLUKA simulation. Apart from the basic structure, any additional elements of the detector should be included manually if necessary. This may include the septa for 2D acquisition mode, shielding elements, etc. The construction of the phantom target, the distribution of radioisotopes, or the beam structure should be further modeled by the user, depending on the specific requirements of the problem under study.

With the purpose of providing the user with a starting ground and facilitating the efficient implementation of a PET scanner, the interface presents several templates of commercial PET detectors, such as Ecat EXACT HR+ (CPS) (124), Ecat HRRT (Siemens) (125), Hi-Rez (Siemens) (126), Allegro (Philips) (127), GE Advance (GEMS) (128), MicroPET P4 (Concorde) (129), MicroPET Focus 220 (Siemens) (130), and Mosaic (Philips) (131). The parameters for such scanners can be further modified before building the geometry, thus serving as a base for detector design and optimizations.

#### 4.3.2 Scoring of Coincident Events

**Figure 18:**
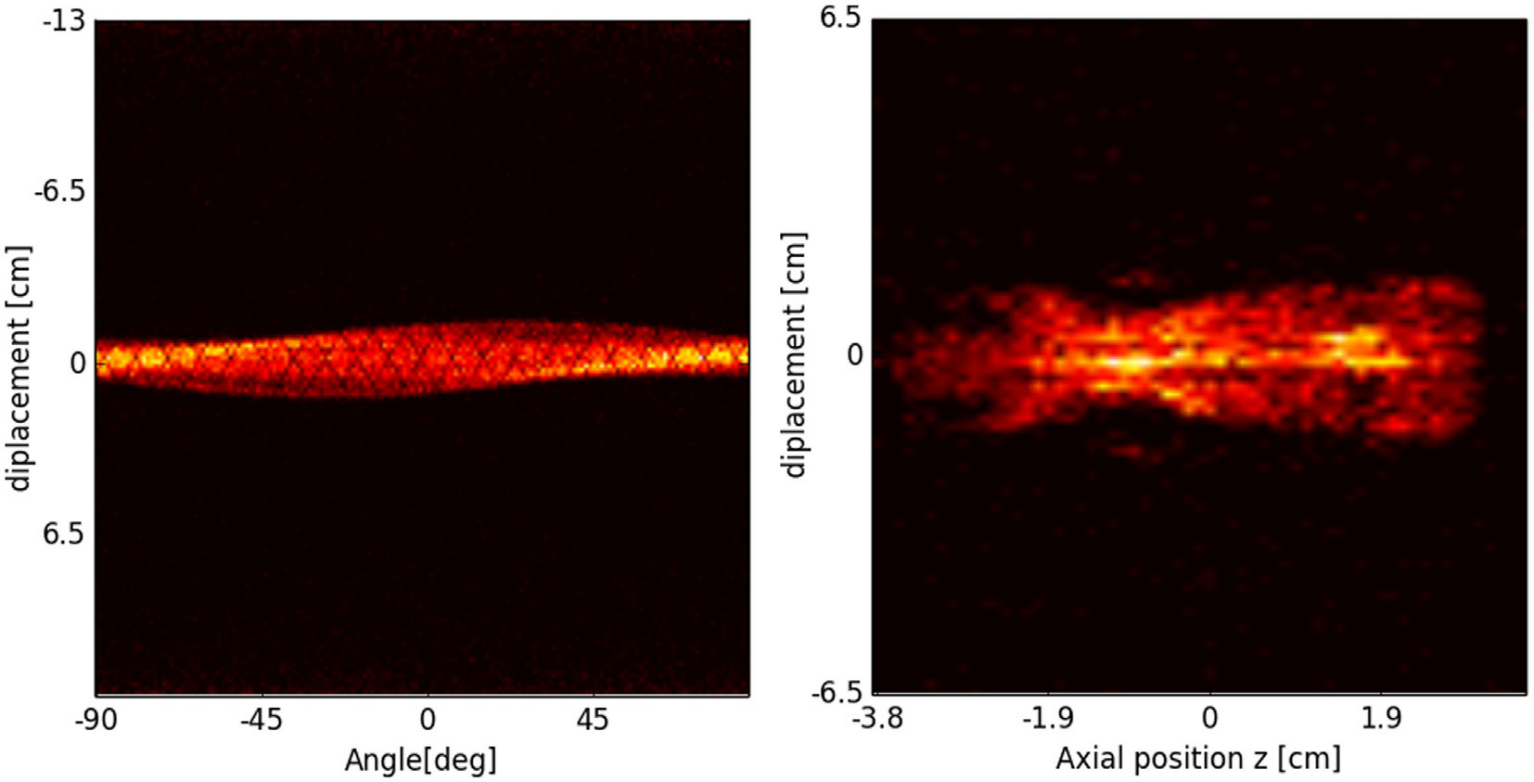
**Sinogram (left) and projection image (right) of the segmented mouse phantom of Figure 17, using a MicroPET P4 scanner**.

In PET, the reconstruction problem consists of obtaining a tomographic slice image from a set of projections. The projections are built by delineating a set of parallel line of responses (LOR), the imaginary line that unites two coincidence events, through the 2D phantom, assigning the integral of all the events registered along each LOR to a single pixel in the projection. Once several projections have been acquired, each of them corresponding to a different angle of the LOR with respect to the phantom, the PET reconstruction of the object can be performed. The set of projections at different angles is called a *sinogram*, which is a linearization of the original image (see Figure 18).

In FLUKA, a collection of scoring routines, complementary to the PET geometry tool, have been implemented, with the goal of acquiring the energy deposition events of the PET scintillators and the subsequent organization of such individual events in coincidence events. The scoring routines are divided in two steps. In a first step, FLUKA simulates the nuclear interactions and tracks the decaying particles through the phantom up to the PET scanner. The portion of energy of such particles deposited in the scintillators is then stored as an individual event, and all the information regarding the event is dumped in a list mode output file. The scoring of individual events can be optimized with several editable parameters:

- the *energy window*, so only particles with a certain amount of deposited energy are scored;
- the *minimum scoring time*, that discards particles arriving at the PET before a time threshold;
- the *time resolution of the detector*;
- the *dead-time of the detector*, which is the time needed for the scintillator to process an event.

Accordingly, one output file with a list of individual events is generated per FLUKA run. In a second step, the set of list mode files is processed, and the coincidence events are produced. The coincidence events output file is organized in *sinograms*, in a *Interfile 3.3* file, a standardized binary intermediate file format for nuclear medicine image data files (132).

The goal is to merge and organize the information produced by the PET data acquisition in a standardized way the user is already familiarized with, and which could be employed with external visualization or reconstruction software. For the sinogram output format, different parameters are available to customize the scoring options: *Arc Correction*, *Maximum Ring Difference (MRD)*, *Number of Segments*, *Span* and *Mashing Factor*. These parameters determine the 2D/3D acquisition mode and its characteristics (see Ref. (133) for further details). The values of these parameters can be conveniently modified by the user.

In addition, simple reconstruction algorithms are planned to be implemented in Flair, so the user can have an image of the object within the same interface. Two algorithms are under development: *2D Filtered Back-Projection* (FBP) and *Maximum-Likelihood Expectation-Maximization* (MLEM). On the one hand, FBP is a simple but fast algorithm, based on the Fourier Transform of the projection and interpolation in Fourier space. The 2D FT transform of the object obtained is then inverted to form the final image. MLEM, on the other hand, is an iterative method that best estimates the reconstruction image by maximizing the likelihood function. It finds the mean number of radioactive decays that better fits the sinogram with the highest likelihood. The output reconstructed image is then stored in USRBIN file, so the result can be further analyzed in Flair.

## 5 Application of the FLUKA Code for Clinical Calculations at HIT and CNAO

The FLUKA code has already been used to support clinical applications prior to the comprehensive extension of the Flair functionality to handle RT objects as described in Section 4.

Dedicated frameworks were implemented in the past at the Heidelberg Ion Beam Therapy Center (HIT, Germany) and the National Center of Oncological Hadrontherapy (CNAO, Italy) providing automated FLUKA MC simulations of clinical treatment plans delivered by actively scanned proton and carbon ion beams (66, 134). Results obtained with these early frameworks have intensively been validated against clinical data and therefore provided a valuable reference for the benchmarking of several RT functionalities during the extension of Flair. The frameworks provide all functionality required for pre-processing of the DICOM RT input data as well as the postprocessing of the FLUKA output. Graphical user interfaces allow to access the fully automated data handling and are realized at HIT within the MeVisLab environment [www.mevislab.de (135)] and at CNAO with Matlab^®^. Physical and RBE-weighted dose distributions are calculated for individual treatment fields and the entire fraction using a global RBE of 1.1 for proton beams and a dedicated implementation of the LEM I framework, which is also used by the treatment planning system (TPS) (4, 75, 136) for carbon ion beams. During the physical and biological calculations dose-to-medium is always converted on-the-fly into dose-to-water, thus providing dose distributions in both formalisms, and dose-averaged LET can optionally be generated for proton beams. In order to assure consistency with the TPS, the FLUKA physics settings are the same as used for the generation of the TPS basic data in water (41, 61, 65). For CT-based calculations, the MC patient model relies on the stoichiometric calibration of Ref. (1, 3) with proper facility and CT-number dependent adjustments of the electromagnetic and nuclear processes as in Ref. (2) for consistency with the CT-range calibration curves used by the TPS for all available CT protocols.

**Figure 19:**
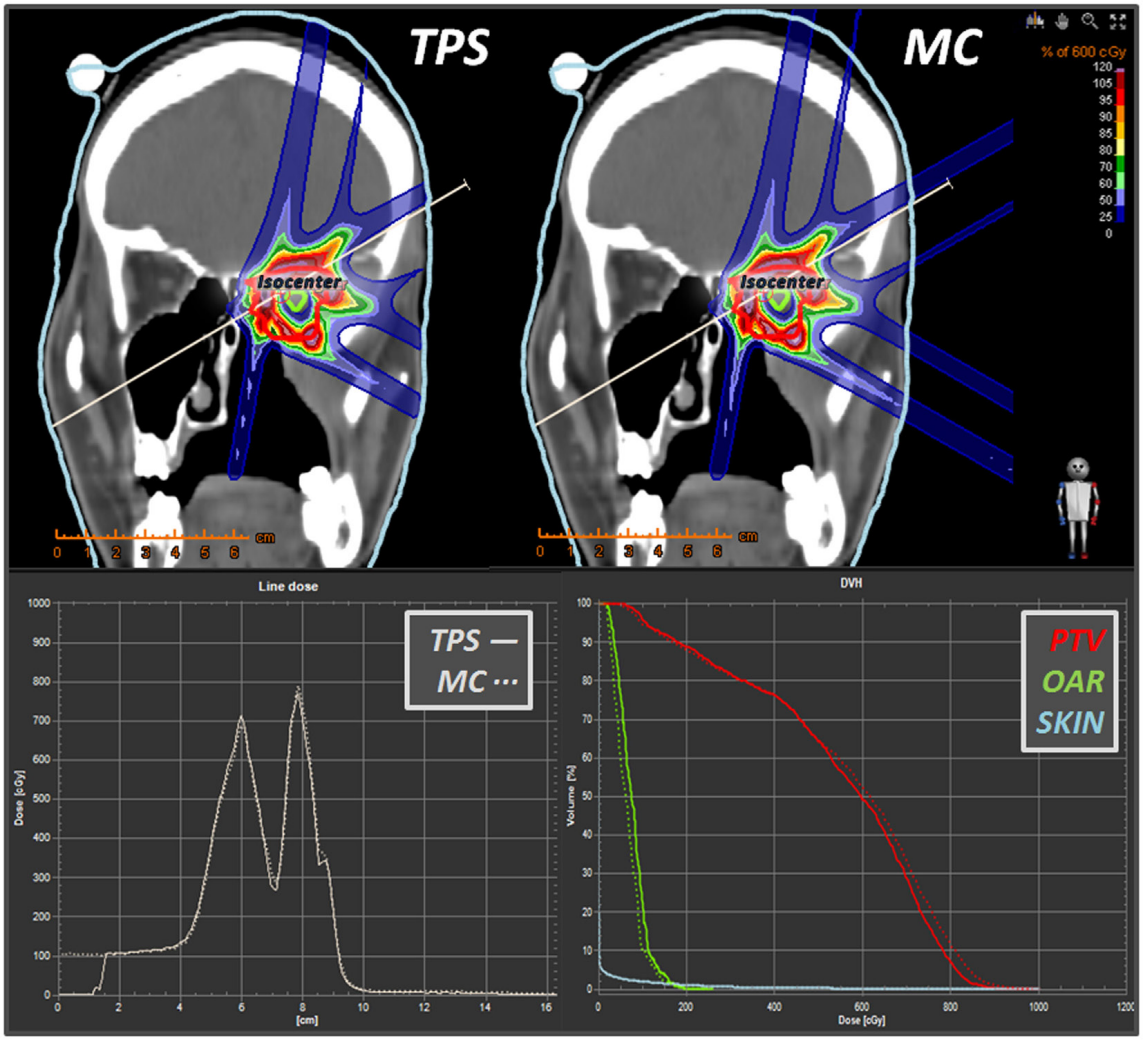
**Some snapshots taken from RayStation^®^ showing a comparison (in coronal view) between a *syngo*^®^ 3-fields carbon ions plan vs. its MC dose forward recalculation**. Lower panels display the line dose and the DVH calculator tools. Differently from the TPS, which displays dose only within the “external-type” structure, the MC dose-to-water scoring is extended to the whole field of view of the CT scan.

The RaySearch RayStation^®^ TPS has been recently (2015) installed at CNAO, and the proton beam line is currently under commissioning. Being specifically thought to provide fast visualization environments and dose statistics tools, a TPS should represent the gold-standard interface to help physicists and physicians to also include MC simulations within the clinical routine. Therefore, in addition to the in-house Matlab^®^ tools, an interface has been developed for converting FLUKA outputs in RTDOSE DICOM files. As an example, Figure 19 reports the physical dose distribution for the study of a 3-fields carbon ions plan (upper panels) for the irradiation of a retro-orbital metastasis. Depth–dose distributions (lower-left panels) and DVHs (lower-right panels) are also displayed. Good agreement has been found between MC and TPS calculated distributions for this challenging case both in terms of profiles and DVHs.

**Figure 20:**
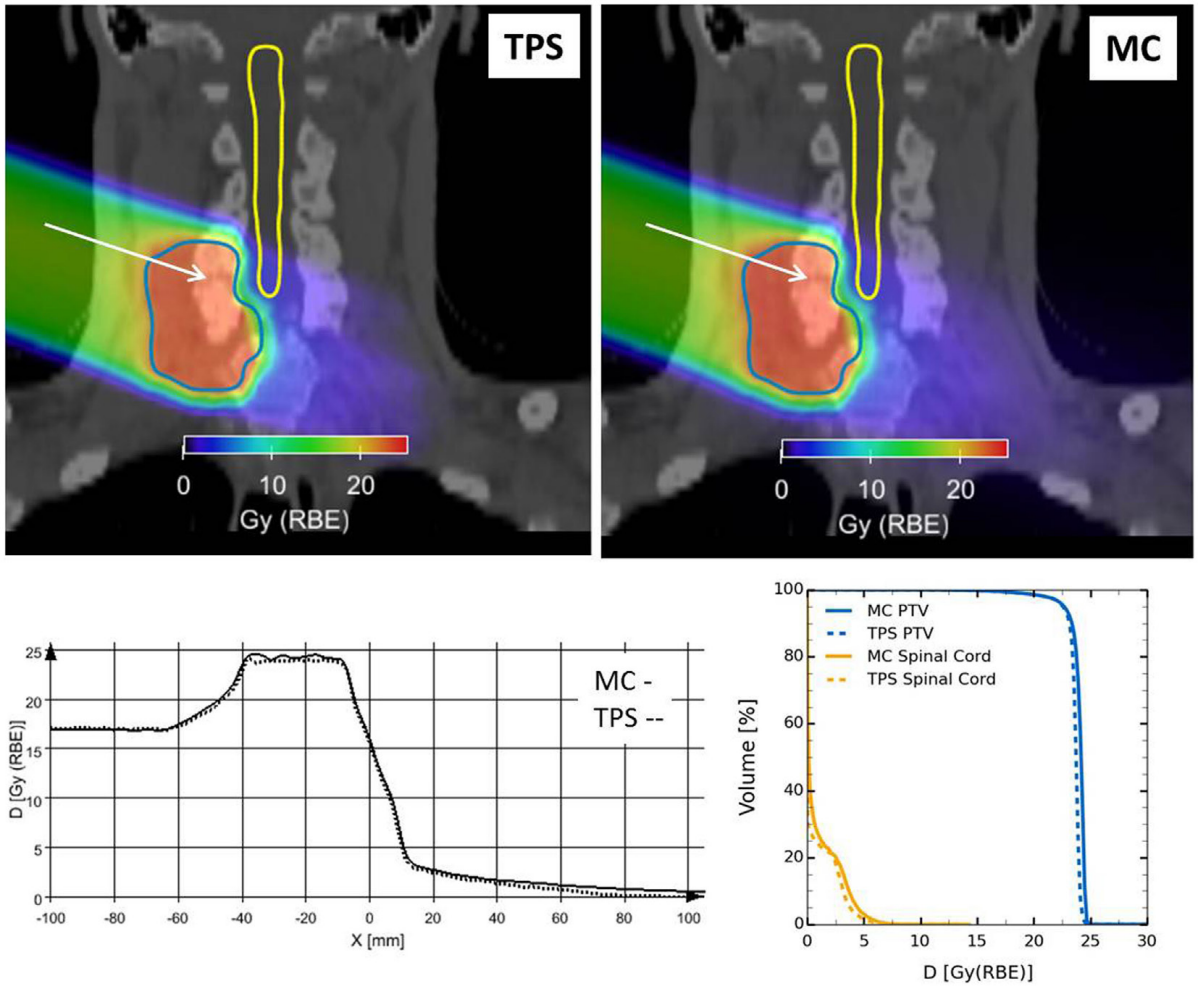
**Clinical-like carbon ion therapy plan delivered to the upper spine region in a single right-lateral field**. Top: overlay views of RBE-weighted dose distributions obtained from the TPS (left) and the FLUKA framework (right) on the treatment planning CT image. Outlined are the PTV and the spinal cord. Bottom: comparison of TPS and MC RBE-weighted dose profiles along the white arrow drawn in the overlay views (left); dose volume histograms for PTV and spinal cord for both TPS and MC RBE-weighted dose distributions (right).

Figure 20 reports the RBE-weighted dose distribution (D_RBE_) of a clinical-like carbon ion therapy plan, delivered to the upper spine region in a single right-lateral field, calculated with the TPS (SIEMENS *syngo*^®^ PT Treatment) and the HIT in-house MC framework (66) described above. Shown are the two-dimensional overlays of D_RBE_ on top of the treatment planning CT image (Figure 20 upper panels), D_RBE_ profiles along a representative line in beams eye view (Figure 20 lower-left panel) and the DVH for the PTV and the relevant organ at risk (spinal cord, Figure 20 lower-right panel). We observe a good agreement between the TPS and the MC calculations, with the MC yielding a slightly higher D_RBE_ level in the target region leading to an increase of D_50_ in the PTV of ≈2%, and of V_10_ in the spinal cord of ≈3%. The slight overestimation of RBE-weighted dose compared to the TPS calculation is attributed to differences in the mixed radiation field description of TPS and MC as discussed in more detail in Ref. (66).

Recently, Flair has been successfully applied at CNAO for performing dose forward calculations with proton beams. In Figure 14, an example of TPS- and MC-calculated dose distributions for a patient-like two fields treatment of a skull-base chondrosarcoma is shown. The satisfactory agreement, as proven by the plotted dose differences (right panel in Figure 14), supports the future usage of Flair as standard re-calculation tool at CNAO.

## 6 Conclusion

The electromagnetic and nuclear models of FLUKA enable to reasonably well reproduce measured depth- and lateral-dose profiles in water for all the spectrum of ions of therapeutic interest, making it the code of choice for generation of TPS input data at leading European centers in Germany and Italy, as well as a valuable tool to support analytical TPS developments of some commercial vendors. In the last years, special efforts have been devoted to improvements of the FLUKA nuclear interaction models, which provide benchmarked and reliable results for interaction cross sections and particle production by proton and ion beams at therapeutic energies. In particular, they allow to treat in a consistent way the transport and interaction of primary particles and all produced fragments, including transport of electromagnetic particles. All reaction generators share the same equilibrium particle emission, thus profiting together of the past and latest developments of the evaporation, fragmentation, and deexcitation models. Low energy nuclear models are of utmost importance for applications to *in vivo* verification techniques. FLUKA is presently able to reproduce within experimental errors the production of *β*^+^ emitters by protons at energies of interest for therapy, and at 25% or better accuracy in the case of carbon projectiles. The newly developed and refined FLUKA models for prompt γ production were shown to reproduce reasonably discrete line cross sections as well as integral energy spectra and yield-vs.-depth data for proton and carbon ion beams. The general trends of the experimental cross-sectional data are consistently reproduced by the models. This includes cross-sectional data of discrete lines for different targets, notably data for carbon, nitrogen, and oxygen nuclei.

The relative shape of photon profiles as a function of depth as well as the absolute photon yield per primary proton and carbon ion (Figure 11) are well reproduced after the last revision of the experimental data (114). The comparisons with the experimental yield-depth data presented here suggest an accuracy of about 15–20% for the prediction of absolute yields. The comparisons of simulated and measured energy spectra for proton beams (Figure 10) showed a very good agreement (mostly within 10%) for photon energies higher than 2 MeV. This is an energy range of interest for prompt-*γ* monitoring and spectroscopy. Progresses on nuclear interaction models are still ongoing, in particular for what concerns low mass ion beams, and a better treatment of spin/parity effects all along the reaction chain.

FLUKA’s physics model reliability is coupled with the versatile features of its Flair graphical interface, creating the necessary input directly from the computed tomography and radiotherapy files. This provides a powerful and user-friendly way to carry out Monte Carlo simulations for medical applications. Flair currently employs a fully functional DICOM CT/MT converter to VOXEL geometry, processing of the RTSTRUCT and RTDOSE modalities, and an automatic PET geometry generator. Work is ongoing on using the RTPLAN and toward the development of a Monte Carlo Treatment Planning System optimizer.

MC dose forward calculation has proven to be a valuable asset to support the development of commercial TP systems in the past. The reported implementations of the FLUKA code in automated workflow environments at HIT and CNAO are intensively used to study the impact of known shortcomings of the analytical approach in particle therapy treatment planning. They provide flexible and robust tools to address daily demands required for high quality patient treatment.

## Author Contributions

GB: promoting the use and development of FLUKA in therapy applications, verifications of physical models, and production of text for the introduction. JB: verification and application of the code in clinical environment, production of text and figures for Section 5. TB: comparison of FLUKA models with dose profiles data, data analysis, and production of text and figures for Section 2. FC: development of FLUKA models for ion interactions in matter and production of text and figures for Sections 2 and 3. MC: development of methods for fluka simulations, analysis of results in prompt photon and PET applications, and critical revision of the manuscript. RA: development of FLUKA models and their application to hadron therapy, and production of text for Section 3. AF: main FLUKA developer, applications to hadronthetrapy and production of text and figures for Sections 2 and 3. PO: comparisons of FLUKA results with data on prompt photons, development of the PET modeler, and production of text and figures for Sections 3 and 4. WK: development of the flair interface for medical applications and production of text and figures for Section 4. GM: applications of FLUKA in clinical environment and production of text and figures for Section 5. AM: development of FLUKA models for ion interactions in matter, implementation of biological effectiveness in FLUKA, clinical applications, and production of text and figures for Sections 2 and 5. KP: pioneering and continuing FLUKA applications to hadrontherapy, data taking and analysis, and production of text and figures for Section 2. PRS: main FLUKA developer, coordination of the manuscript preparation, and production of text and figures for Sections 2 and 3. PS: developments of nuclear properties database for FLUKA, comparisons of FLUKA results with data, and general editing of the manuscript. TT: data taking, analysis and comparisons with MonteCarlo of dose-depth distributions, and production of text and figures for Section 2. VV: main author of Flair, applications to hadrontherapy, and production of text for Section 4.

## Conflict of Interest Statement

The authors declare that the research was conducted in the absence of any commercial or financial relationships that could be construed as a potential conflict of interest. The reviewer FB declared a shared affiliation, though no other collaboration, with the authors GB and PS to the handling Editor, who ensured that the process nevertheless met the standards of a fair and objective review.
